# Anonymization and visualization of health data and biomarkers

**DOI:** 10.1038/s41746-026-02662-x

**Published:** 2026-05-02

**Authors:** Minh H. Vu, Daniel Edler, Carl Wibom, Martin Rosvall, Beatrice Melin

**Affiliations:** 1 Department of Diagnostics and Intervention, Umeå University, Umeå, Sweden; 2 IceLab, Department of Physics, Umeå University, Umeå, Sweden

**Keywords:** Computational biology and bioinformatics, Health care, Mathematics and computing, Medical research

## Abstract

Access to large, diverse biomedical datasets is critical for advancing medical research, yet privacy regulations severely restrict data sharing. We present an end-to-end framework for privacy-preserving health data synthesis that integrates advanced deep generative models (DGMs) with robust preprocessing, formal differential privacy (DP) training for select DGMs, empirical privacy risk evaluation, data-sufficiency analysis, domain-guided quality control, and biobank visualization tools. Released as open-source containerized software, the framework ensures reproducible deployment while preserving statistical fidelity, machine learning (ML) utility, and privacy guarantees. Empirical evaluations across diverse biobank datasets demonstrate that TabSyn—a transformer-based diffusion model–combined with our correlation—and distribution-aware CorrDst loss function achieves superior performance balancing fidelity, privacy, and computational efficiency. The tailored preprocessing pipeline effectively handles high missingness rates, substantially improving distributional accuracy and clinical plausibility. Across 26 biobank datasets spanning three regulatory levels, the framework shows that TabSyn with correlation- and distribution-aware loss function consistently achieves superior performance in terms of fidelity, privacy, and computational efficiency.

## Introduction

Breakthroughs in medicine depend on access to large, diverse, and standardized datasets^1–6^. These datasets allow researchers to identify patterns, predict outcomes, and tailor treatments^7–9^. Yet much of this valuable information remains locked away. To protect patient integrity and comply with the general data protection regulation (GDPR) regulations, institutions restrict access to sensitive data. These restrictions vary between institutions, making it difficult to pool datasets^10–13^. Large biobank initiatives, such as the UK Biobank, offer structured access to superficial health data that lack opportunities for subgroup validation and suffer from selection bias that limits generalizability^14^.

Less-biased datasets that clear legal and ethical hurdles often contain missing values, forcing researchers to rely on imperfect imputation methods^15–17^. Small sample sizes compound these problems. These constraints hit rare disease research hardest, where individual cohorts are too small to support meaningful analysis. Multi-institutional retrospective registry studies, particularly those linked to stored biological samples, represent a gold mine of untapped information^18^. Despite their potential, these resources remain locked behind legal, ethical, and technical barriers.

Privacy-preserving synthetic data generation offers a potential solution^19,20^. Deep generative models (DGMs), including generative adversarial networks (GANs), variational auto-encoders (VAEs), and diffusion models (DMs)^21–25^, can produce synthetic datasets that approximate real-world distributions while protecting patient privacy. However, these models face fundamental challenges that limit their application in medicine. Many distort relationships between variables in clinical datasets^26–31^, such as age, BMI, and disease status, producing synthetic records that are statistically inconsistent or clinically implausible^24,32–34^. Other models rely on heuristic privacy protections without formal differential privacy (DP) guarantees. The generated data may be either too noisy to be useful or too realistic to be safe.

Beyond technical accuracy and privacy, practical barriers remain. Poor documentation, unclear dependencies, and complex installation make many pipelines difficult to use. Others require expert configuration: manual tuning of loss functions or preprocessing steps unsuitable for mixed-type, high-dimensional health data^7,35–37^. High missingness in laboratory results, skewed vital sign distributions, or essential logical constraints, such as ensuring that pregnancy only occurs in female patients, cause many models to fail^17,38–40^. These failures limit how researchers can integrate synthetic data into standard analytical workflows.

To address these challenges, we develop an end-to-end framework that integrates advanced DGMs with tools for synthesizing and visualizing sensitive health data. The framework preserves statistical integrity while ensuring privacy and supporting integration with standard analytical workflows. We release the entire framework as open-source software, distributed with containerization to ensure reproducibility and straightforward deployment across institutional environments. To ensure that the framework meets ethical and legal standards, we secured approval through an amendment to the regional ethics committee and conducted a comprehensive risk and consequence assessment.

The principal contributions of this work are threefold. First, we introduce a privacy-aware synthetic data generation framework ready to use. The software is open source and packed as a Docker container. A single configuration file controls dataset attributes, preprocessing rules, and privacy constraints.

Second, we develop a data-handling and evaluation framework tailored to biomedical applications. This framework includes four components: (1) A robust preprocessing pipeline for health datasets, which is broadly applicable to sensitive datasets and handles high missingness rates. (2) Formal DP training for select DGMs grounded in our prior work^31^ and empirical privacy risk evaluation, (3) a systematic data-sufficiency analysis determines the minimum sample sizes required for reliable generative model training. (4) Postprocessing and rejection sampling mechanisms enforce expert-defined correlations and logical constraints, ensuring that synthetic outputs maintain both statistical fidelity and clinical plausibility. These components provide practical guidance for preparing, evaluating, and deploying DGMs in privacy-constrained biomedical settings.

Third, we implement visualization tools for anonymized health data. These tools integrate with the synthetic-data pipeline to provide privacy-aware visual summaries. Researchers and clinicians can explore and interpret health data without compromising privacy.

To validate the framework, we apply it to high-dimensional health datasets with substantial missing values. The generated synthetic data preserve key distributional properties and relationships between variables while meeting privacy constraints. In addition, domain-informed quality control measures are implemented to ensure clinical plausibility and data integrity. These assessments confirmed that the resulting synthetic datasets achieve high fidelity and utility while maintaining strict privacy protection and biological realism. Overall, our results highlight that careful preprocessing, rigorous privacy evaluation, explicit data-sufficiency assessment, and domain-guided quality control are essential for generating reliable and privacy-preserving synthetic datasets. The open-source implementation and visualization tools aim to accelerate privacy-preserving data sharing and support reproducible healthcare research.

## Results

### Study design and participant selection

The PREDICT study integrates six cohorts from Västerbotten County, Sweden, including the Västerbotten Intervention Project, which has collected health data and stored blood samples since 1990 and continued to recruit participants. Individuals selected for inclusion in PREDICT were chosen on a convenience basis, focusing on those who had provided a first blood sample by the end of 2010, to allow for adequate follow-up and to enrich the cohort with individuals aged 50 years or older. Ethical approval included a waiver to allow the inclusion of deceased individuals. Following the distribution of invitation letters during 2021–2022 and subsequent data entry and cleaning, the final PREDICT dataset comprises 50,274 individuals.

To establish a system for synthetic data generation and visualization, we submitted an amendment to the regional ethics committee and conducted a comprehensive risk and consequence assessment. This was required to enable data sharing with Sweden’s national research platform and necessitated a formal personal data assistant agreement. The full process of documentation, ethical review, and approval took approximately nine months. Following approval, we implemented the necessary preprocessing steps to prepare the data for testing with synthetic data generation algorithms. This process illustrated the multiple, complex, and time-consuming steps required to make real data ready for analysis. It underscored the potential value of using synthetic data, provided it adequately captured the characteristics of the real data, as a safer and more efficient alternative for enabling meaningful research.

The PREDICT cohort contains individuals from the Norrbotten and Västerbotten regions. This resource integrates multiple data modalities, including over 927,000 entries from patient journals, more than 22,000 metabolomics samples, and upwards of 89,000 questionnaire responses. The dataset further contains detailed information on demographics, anthropometric and blood measurements, cardiovascular events, diabetes status, and cancer diagnoses. These diverse data sources collectively form the foundation for downstream analyses and synthetic data generation.

The datasets were organized into three levels: Level 1, involving the removal of sensitive information prior to any GDPR approval; Level 2, conducted under GDPR-compliant approval but based on early-stage and incomplete data; and Level 3, also under GDPR-compliant approval, which provided complete and fully integrated data.

In the First Level, strict legal restrictions mandated the removal of direct identifiers and the generalization of sensitive variables such as age. Only anonymized cancer registration data were used, resulting in seven derived datasets. These included vital status, death records, and multiple ICD-coded cancer classifications (ICD-7, ICD-9, ICD-O2, and ICD-O3).

The Second Level enabled access to additional sensitive data under controlled conditions, producing eight new datasets that expanded beyond cancer registry information to include patient journals, questionnaires, and 25% of the available metabolomics data. These datasets focused on cancer-specific subgroups (prostate, breast, colorectal, urothelial and kidney, lung, pancreatic, and hematological cancers) as well as a dedicated metabolomics cohort.

Finally, the Third Level incorporated the full metabolomics collection (100%) and applied final preprocessing steps. Eleven new datasets were created at this stage, including both a combined cancer cohort and multiple disease-specific subsets, providing enriched granularity for analysis.

**Fig. 1:**
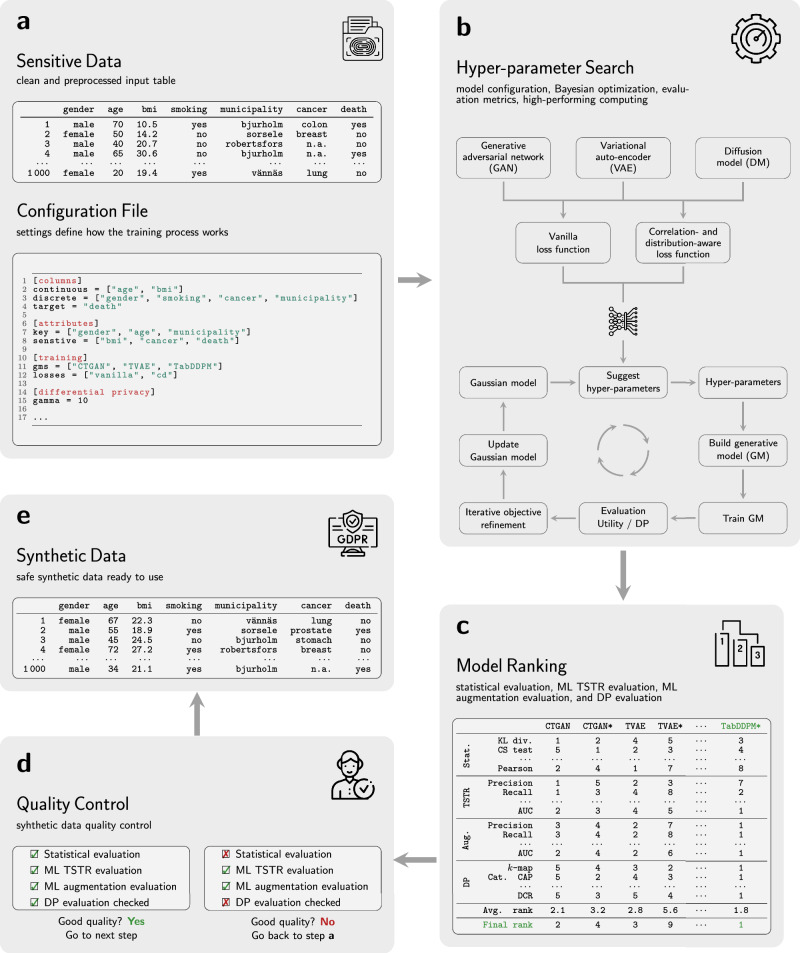
Overview of the PREDICT cohort, dataset hierarchy, and experimental setup. **a** PREDICT: cohort of 50,274 individuals in northern Sweden with patient journals (more than 927,000 entries), metabolomics (more than 22,000 samples), and questionnaires (more than 89,000 responses), covering demographics, health metrics, blood measures, cardiovascular data, diabetes, and cancer. **b** First level: seven anonymized cancer datasets pre-GDPR. **c** Second level: eight datasets post-GDPR, early-stage/incomplete. **d** Third level: 11 complete datasets with full metabolomics. **e** Training: 80/10/10 split; Intel Xeon, NVIDIA A100, 256 GB RAM; GPU runtime around 700 GPU days.

Figure 1 shows the structure and composition of the datasets across these three levels.

### Health data anonymization framework

**Fig. 2:**
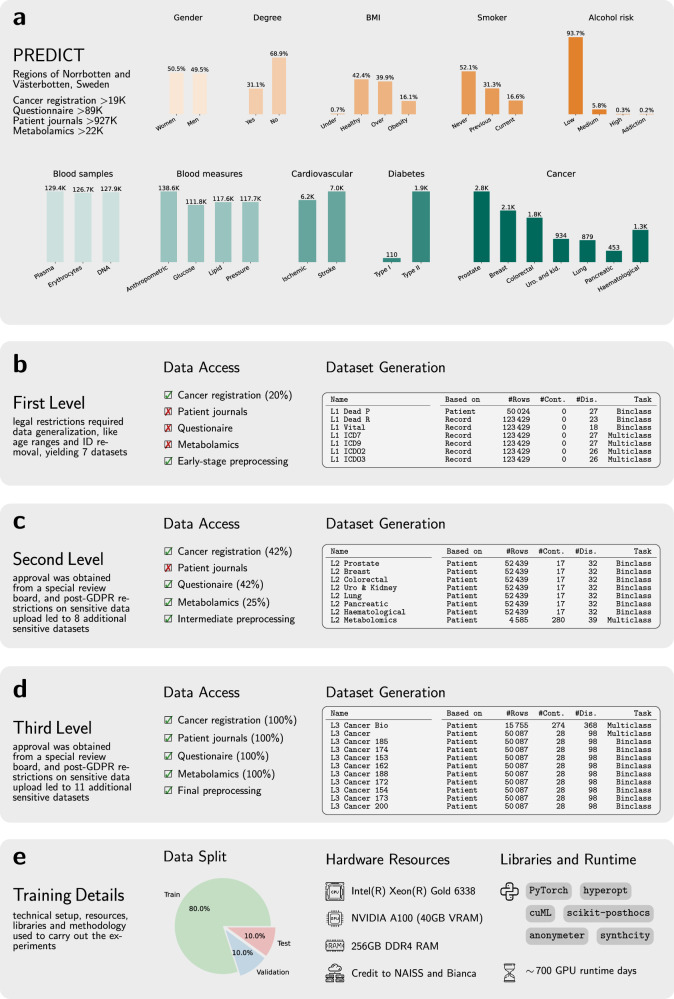
End-to-end framework for privacy-preserving synthetic health data generation. a^*^ Sensitive data: input table and configuration file with attributes, rules, and privacy constraints. **b** Model training: generative models (generative adversarial networks (GANs), variational auto-encoders (VAEs), diffusion models (DMs)) are optimized with Bayesian search to balance utility and privacy. **c** Model ranking: candidates are scored by statistical, ML, and DP metrics. **d** Quality control: automated tests and expert review validate fidelity, utility, and privacy; failures loop back to (**a**). **e** Synthetic data: final datasets preserve key statistics and privacy, enabling secure sharing. ^*^Fictive data; for illustrative purposes only.

Figure 2 illustrates the health data anonymization workflow, which consists of five interconnected stages (see Supplementary Material Fig. S1 for more details). The process began with a curated and preprocessed input table derived from sensitive sources such as clinical records, questionnaires, or registries. A configuration file accompanied the data, specifying attributes, preprocessing rules, and explicit privacy constraints, thereby embedding legal and ethical safeguards into the pipeline from the outset.

In the second stage, DGMs including CTGAN^26^, CTAB-GAN^27^, DP-CGANS^28^, CopulaGAN^26^, TVAE^26^, TabDDPM^29^, and TabSyn^30^ were trained under two alternative loss functions: a standard baseline (BaseFn) and a correlation- and distribution-aware loss (CorrDst^31^). To balance data utility with privacy, hyperparameters were optimized via iterative objective refinement Bayesian optimization (IORBO) (see “Relation to prior work”). This systematic exploration identified model configurations that jointly maximized statistical fidelity, machine learning (ML) utility, and privacy guarantees.

The third stage involved rigorous evaluation and ranking of candidate models across multiple dimensions. Statistical similarity metrics quantified fidelity to the original data distribution, while ML-based evaluations, including Train-Synthetic-Test-Real (TSTR) performance and augmentation benchmarks, assessed the transferability of synthetic data to downstream tasks (see “Relation to prior work”). In parallel, privacy guarantees such as DP were computed (see “Differential privacy evaluation”). Scores from these criteria were aggregated to select the most reliable model for synthetic data generation.

In the fourth stage, the chosen synthetic datasets underwent strict quality control (see “Quality control measures”). Both automated assessments and expert review were employed to verify fidelity, utility, and privacy. This “human-in-the-loop” step meant that domain experts-such as clinicians and data specialists-directly evaluated whether the generated data were realistic, clinically meaningful, and ethically appropriate. If requirements were not met, the workflow iterated back to earlier stages for refinement.

Finally, once all evaluations were passed, the synthetic dataset was released. The resulting data preserved key statistical properties of the original while ensuring strong privacy guarantees, enabling safe, reproducible, and ethically responsible data sharing for research and analysis.

### Comparative evaluation of model rankings

**Fig. 3:**
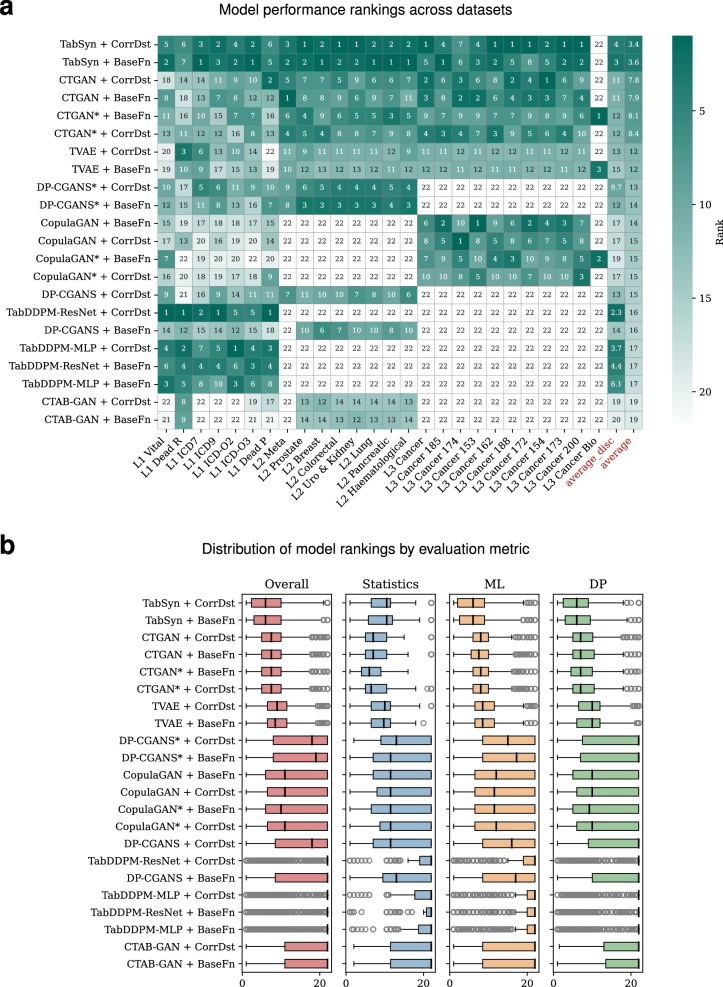
Comprehensive performance evaluation of generative models across biomedical datasets. **a** Heatmap of model performance rankings across all 26 biomedical datasets, with models on the y-axis and datasets on the x-axis. Color intensity refl ects rank, where darker shades indicate lower (better) rankings and rank 22 denotes model failure. The rightmost columns show average rank and average rank on discrete datasets. **b** Boxplots showing the distribution of model rankings across four evaluation categories (Overall, Statistics, ML utility, and DP) for each generative model, illustrating both median performance and variability.

Figure 3a shows a heatmap ranking the performance of DGMs across biomedical datasets, with models on the *y*-axis and datasets on the *x*-axis. Rankings specific to “Statistical,” “Machine Learning,” and “Differential Privacy” are shown in Supplementary Material Figs. S2 and S3a. Datasets were grouped into three levels: Level 1 (sensitive information removed), Level 2 (post-GDPR approval, incomplete data), and Level 3 (post-GDPR approval, complete data). An “average” column highlights the mean rank per model as a summary of overall performance.

Across the ranking scale, where rank 1 was best and rank 22 worst, TabSyn + CorrDst and TabSyn + BaseFn consistently appeared near the top, reflecting robustness across heterogeneous datasets. In contrast, models such as CTAB-GAN + CorrDst frequently fell to the lower ranks, suggesting difficulty handling dataset complexity. Blank cells (rank 22) denoted failures from GPU out-of-memory (OOM), runtime errors, or invalid outputs, as further detailed in Supplementary Material Fig. S3b and Runtime Efficiency and Model Reliability.

Transformer- and diffusion-based approaches, particularly the TabSyn family, clearly outperformed traditional GAN-based models. With average ranks as low as 3.4, TabSyn variants proved resilient across Levels 1–3 datasets, indicating a strong capacity to capture complex correlations in medical data. By contrast, CTAB-GAN and some CopulaGAN variants yielded average ranks above 15 with frequent failures in Level 3, underscoring challenges in scaling to high-dimensional or sparse data.

The “average_disc” column, computed for Level 1 categorical datasets, highlighted the advantage of diffusion and transformer models for discrete variables. TabDDPM-ResNet + CorrDst (average rank 2.3) and TabSyn variants (3.0–4.0) dominated, while traditional GANs-particularly CTAB-GAN-performed poorly (average > 19).

Figure 3 complements the heatmap with boxplots summarizing four evaluation categories: Overall, Statistical, ML utility, and DP. TabSyn models achieved consistently high ranks and narrow spreads across categories, reflecting both accuracy and stability. In contrast, CTAB-GAN and TabDDPM showed weaker and less reliable outcomes. CorrDst further boosted performance by improving correlation preservation without compromising privacy, benefiting models such as TabSyn + CorrDst and TabDDPM-ResNet + CorrDst.

Taken together, the heatmap and boxplots provide a comprehensive view of model performance. The heatmap highlights generalization patterns, while the boxplots reveal stability across evaluation categories. Clear trends emerged: CTGAN offered strong statistical fidelity but limited overall utility, CopulaGAN and CTAB-GAN underperformed broadly, and privacy-focused models like DP-CGANS* + CorrDst traded utility for DP. In contrast, TabSyn + CorrDst achieved the most balanced results across all evaluations.

### Runtime efficiency and model reliability

Figure S3b in the Supplementary Material shows runtime and failure modes across datasets. Execution times were categorized as fast (<2 h), moderate (2–4 h), acceptable (4–8 h), slow (8–16 h), and infeasible (>16 h), with additional categories for OOM, timeouts, and invalid outputs. Level 1 datasets ran quickly, while Level 3 and disease-specific datasets had longer runtimes and more failures.

Among evaluated DGMs, TabSyn variants consistently achieved fast to moderate runtimes, with both BaseFn and CorrDst contributing to stable performance. CTGAN and TVAE were generally acceptable, while TabDDPM was occasionally faster due to skipping VAE pretraining. Models with ResNet backbones required more resources than those with MLP. In contrast, DP-CGANS and CTAB-GAN frequently exceeded runtime limits or encountered OOM errors, particularly on larger datasets.

Failure modes highlighted model reliability differences. OOM errors occurred when GPU memory was insufficient, timeouts when training exceeded 7 days, and invalid outputs when models produced degenerate results. The proposed preprocessing pipeline improved stability; for example, CopulaGAN succeeded on Level 3 only after preprocessing. Across models, CorrDst also enhanced runtime stability.

These results indicate that TabSyn, CTGAN, and TVAE provide the most reliable runtimes. Combined with the proposed preprocessing pipeline and CorrDst loss, they are recommended for large-scale biomedical synthesis, whereas other models often failed due to inefficiency or instability.

### Comparative analysis of preprocessing methods

This section compares the standard preprocessing approach used in prior work^31^ with the proposed pipeline (see Preprocessing Pipeline for Model Training). Unlike general-purpose datasets, biomedical data frequently exhibit substantial missingness, necessitating robust preprocessing to generate reliable synthetic samples. In the sensitive biomedical datasets analyzed here, missing values ranged from 0.4 to 90%.

**Fig. 4:**
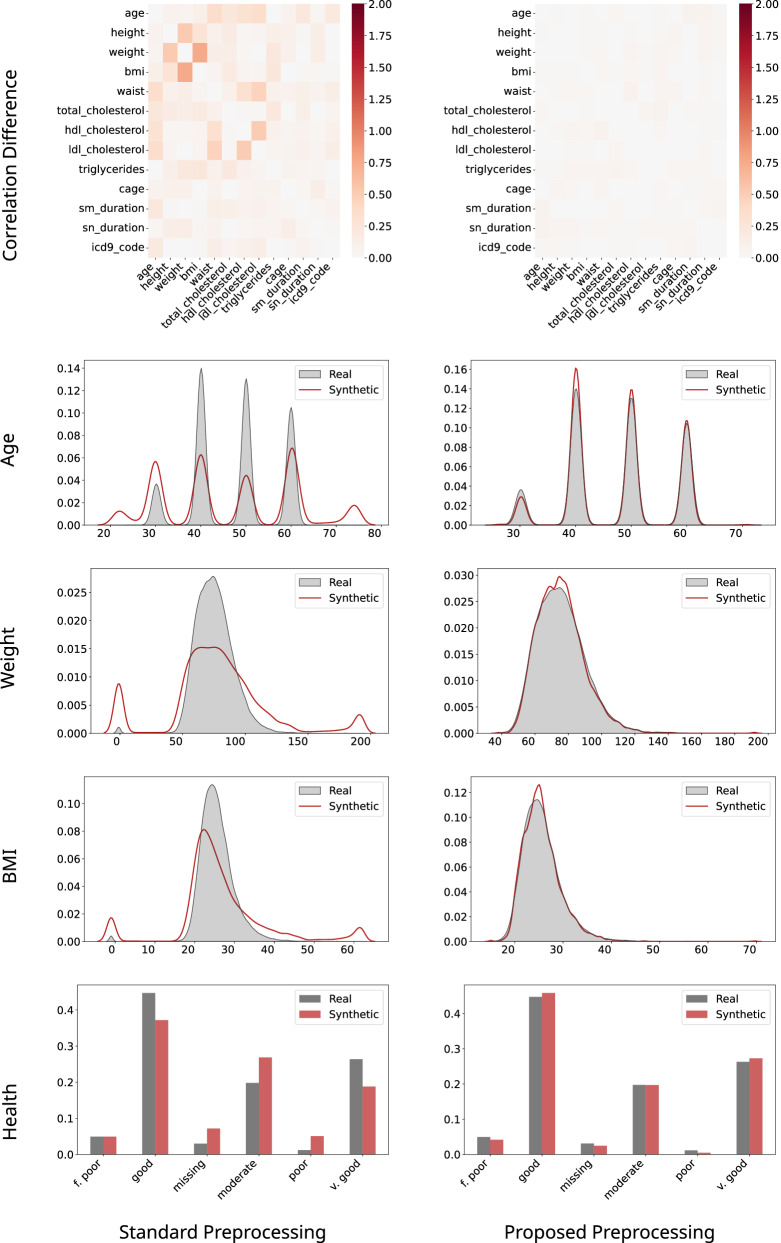
Effect of preprocessing pipeline on synthetic data quality for the L3 Cancer dataset. Impact of preprocessing on synthetic data quality for the L3 Cancer dataset using identical TabSyn configurations. Standard preprocessing with simple missing value replacement **a**, produces substantial correlation deviations and distribution artifacts. The proposed pipeline **b**, employing structured imputation, quantile transformations, and missingness indicators, achieves closer alignment with real data across correlations and marginal distributions.

A direct comparison between standard preprocessing (Fig. 4a) and the proposed pipeline (Fig. 4b) shows clear improvements in synthetic data quality for the L3 Cancer dataset using identical TabSyn configurations. Correlation-difference heatmaps revealed that standard preprocessing introduced large deviations across key variable pairs, such as “height” vs. “weight,” “weight” vs. “BMI,” and “waist” vs. cholesterol measures. In contrast, the proposed pipeline preserved multivariate relationships, with only minor residual differences, reflecting improved statistical fidelity and retention of clinically meaningful dependencies.

Kernel density estimate (KDE) plots for “age,” “weight,” and “BMI” further highlighted these benefits. Standard preprocessing produced spurious peaks and unrealistic extremes (e.g., age above 70, weight exceeding 200 kg, BMI greater than 60), whereas the proposed pipeline yielded distributions closely matching the real data. Similarly, the “health” categorical variable was distorted under standard preprocessing, over-representing “missing” and under-representing “good” categories; these distortions were corrected by the proposed method.

These improvements resulted from the systematic treatment of missingness. Rather than relying on constant-value imputation (e.g., −1) and naive one-hot encoding, the pipeline imputed continuous and date variables with medians, applied quantile transformations to stabilize scales and mitigate outliers, and encoded categorical variables with explicit missingness indicators. Its bi-directional design supported full reconstruction of original data types and structures, ensuring interpretability and reproducibility. Consequently, synthetic datasets generated using this pipeline closely aligned with real data in both correlations and marginal distributions, preserving signals essential for downstream biomedical analyses.

### Qualitative result

In this section, we present a qualitative comparison of synthetic data generated by TabSyn, CTGAN, and TVAE on the L3 Cancer dataset processed with our proposed preprocessing pipeline (see “Preprocessing pipeline for model training”). The dataset featured high missingness and complex clinical variables, providing a challenging benchmark for DGMs.

**Fig. 5:**
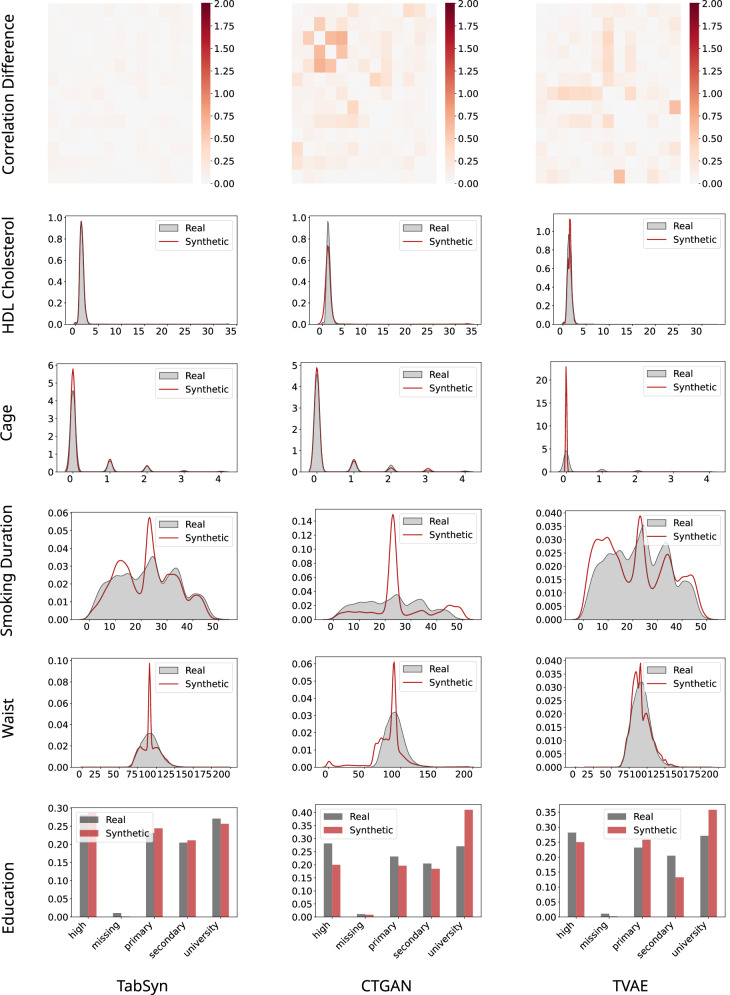
Qualitative comparison of synthetic data generated by TabSyn, CTGAN, and TVAE on the L3 Cancer dataset. Qualitative comparison using correlation-difference heatmaps and kernel density estimate (KDE) plots. TabSyn best preserves correlation structure and categorical distributions, TVAE shows local strengths (e.g., “Waist”), and CTGAN exhibits the largest deviations and artifacts, indicating weaker modeling of complex, high-missingness biomedical data.

Correlation-difference heatmaps in the first row (Fig. 5), showing absolute differences between synthetic and real data correlations, indicated that TabSyn maintained the strongest multivariate dependencies, TVAE performed moderately, and CTGAN exhibited widespread mismatches. These results emphasized the importance of model design in capturing clinically meaningful inter-variable relationships.

KDE plots for selected continuous variables (e.g., “HDL cholesterol,” “Smoking duration,” “Waist”) revealed further contrasts. TabSyn generally captured distribution shapes with minimal distortion, TVAE performed well on specific features like “Waist,” and CTGAN introduced artifacts and failed to reproduce multimodal structures accurately.

For categorical variables such as “Education,” TabSyn closely mirrored real distributions, TVAE tended to overcompensate, and CTGAN under-represented most categories. These differences highlighted the impact of model stability and training robustness on categorical fidelity.

Overall, TabSyn consistently aligned with both univariate and multivariate properties, TVAE showed localized strengths, and CTGAN generally underperformed. These qualitative assessments demonstrated the value of structured preprocessing and architectures capable of modeling complex biomedical data, establishing TabSyn as the most reliable performer in this setting.

For quantitative evidence, additional results on ML TSTR, data augmentation, and DP are reported in Tables S1 and S2 in the Supplementary Material, which provide direct numerical comparisons of synthetic data generated by TabSyn, CTGAN, and TVAE on the L3 Cancer dataset processed with our proposed preprocessing pipeline.

### Evaluating minimum data requirements for synthetic data generation

**Fig. 6:**
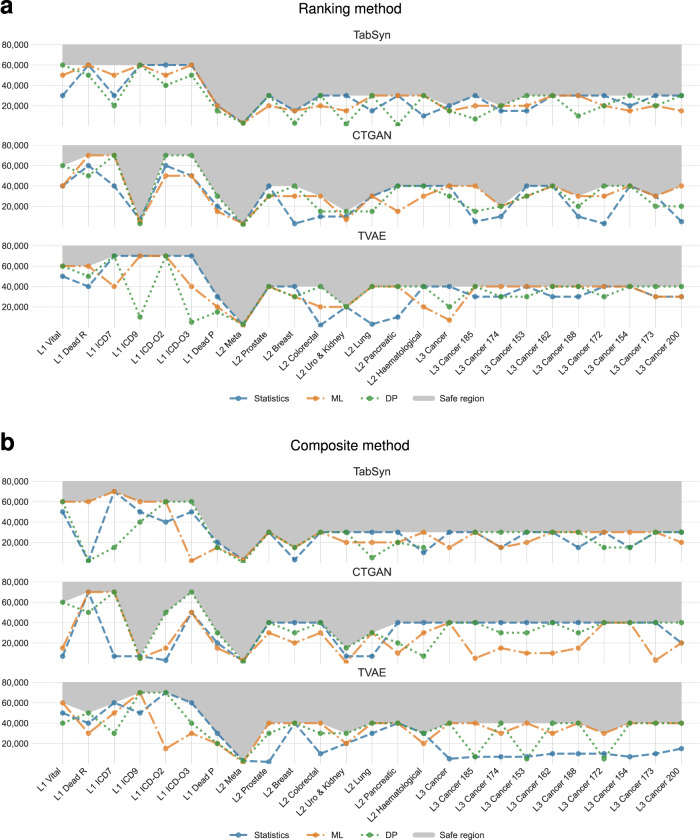
Data sufficiency analysis comparing ranking and composite methods across generative models and evaluation metrics. **a** Ranking-based method showing aggregated performance ranks across generative models and evaluation metrics as a function of training sample size, with the elbow point indicating the minimum sufficient sample size. **b** Composite score-based method consolidating statistical, ML, and DP scores into a single normalized curve per model, with the elbow point identifying the sample size threshold where performance plateaus.

Figure 6 shows a systematic evaluation of the minimum sample sizes necessary for training DGMs (CTGAN, TVAE, TabSyn) to produce high-fidelity synthetic biomedical data. Incremental subsets of the original datasets, ranging from 1000 to 70,000 records, were sampled to evaluate performance across varying data volumes. For each subset, hyper-parameter optimization using IORBO was performed, and the resulting models were assessed on statistical, ML, and DP metrics. To summarize performance, ranks across models and metrics were aggregated using the Nemenyi test, and a smoothing spline combined with the KneeLocator algorithm^41^ identified the elbow point where performance stabilized. This elbow represented the minimum number of samples required to generate synthetic datasets that were both reliable and privacy-preserving.

The thresholds revealed consistent and interpretable patterns across models and dataset levels. TabSyn consistently required fewer samples—approximately 30,000—than CTGAN or TVAE, highlighting the efficiency of its transformer-based architecture, especially in scenarios with limited data.

Interestingly, Level 1 datasets, which were fully discrete and structurally simpler, required 40,000–60,000 samples, whereas Levels 2 and 3 stabilized with 20,000–40,000 samples. This counterintuitive trend may be related to the limited feature diversity in Level 1 datasets, which restricts the variety of observable feature combinations and may require more samples to accurately estimate the joint distribution. In addition, datasets dominated by low-cardinality discrete variables may be more sensitive to noise introduced by differential privacy mechanisms compared to datasets containing richer continuous feature spaces. These factors together may contribute to the higher observed data requirements for Level 1 datasets.

Metric-specific analyses further illuminated these patterns: statistical fidelity generally stabilized at smaller sample sizes, whereas DP metrics consistently drove thresholds higher, emphasizing the inherent trade-off between utility and privacy. Comparisons between the ranking and composite methods demonstrated strong agreement, with differences of only 5000–10,000 samples, reinforcing the reliability of the identified thresholds. Overall, these results provide actionable guidance for determining sample-size requirements, highlighting that dataset complexity, generative architecture, and privacy considerations jointly determine the efficiency of synthetic data generation in biomedical contexts.

### Quality control measures

To assess the clinical plausibility and utility of the synthetic data, we performed domain-informed validity checks grounded in established medical knowledge. These included verifying expected associations, such as sex-specific cancer patterns and known risk factors, using frequency tables and logistic regression. Implausible findings were flagged and fed into the refinement of the postprocessing stage; for example, occurrences of biologically impossible cases (such as females assigned “prostate cancer”) informed the rejection rules applied after generation. A dedicated postprocessing step using rejection sampling (see Postprocessing and Rejection Sampling) was then employed to enforce these expert-defined constraints, ensuring that the final synthetic data maintained clinical realism.

**Fig. 7:**
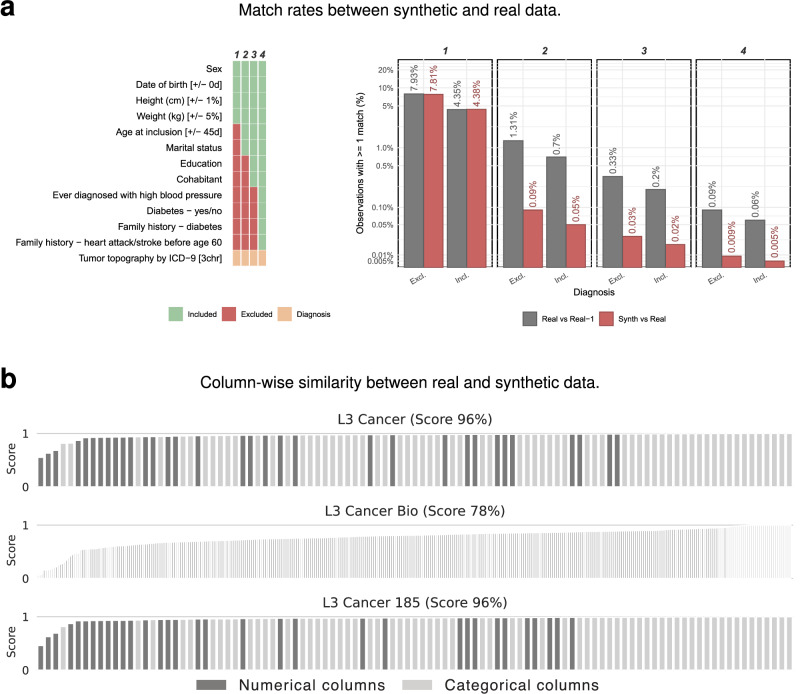
Synthetic versus real data comparison across match rates and column-wise distributional similarity. **a** Match rates between synthetic and real data across four variable configurations (1–4). Each bar shows the proportion of observations that matched at least one real record on all selected variables. Synthetic-to-real match rates are consistently lower than real-to-real match rates (excluding self-matches), indicating that the synthetic data does not leak individual-level information from the original dataset. **b** Column shapes: statistical similarity per column between synthetic and real data for three Level 3 datasets. Numerical columns are scored using the Kolmogorov–Smirnov statistic and categorical columns with the total variation distance.

Beyond clinical plausibility, we evaluated privacy risks using complementary approaches. First, we applied a heuristic matching approach that measured how closely synthetic records resembled real ones across four sets of pseudo-identifiable variables. To contextualize these results, we applied the same matching logic within the real dataset while excluding matches to the same individual. Synthetic data consistently showed lower match rates than real data (Fig. 7a), indicating no evidence of individual-level leakage.

We further evaluated privacy and utility using the Disclosure Protection score from the Synthetic Data Metrics (SDMetrics) library^37^, summarizing results across biomedical datasets in Table S3 in the Supplementary Material. The SDMetrics Diagnostic Report confirmed alignment between synthetic and real data in terms of data structure and variable ranges (Data Validity), while the Quality Report assessed statistical similarity in univariate distributions (Column Shapes) and pairwise dependencies (Column Pair Trends). Overall, the synthetic datasets showed strong statistical fidelity, with only a small number of columns exhibiting poor similarity (Fig. 7b). As expected, performance degraded for the L3 Cancer Bio dataset, where the substantially higher dimensionality (642 columns and 15,755 rows) compared to the L3 Cancer dataset (126 columns and 50,087 rows) presents a more challenging modeling task. This imbalance reduced the ability to accurately capture individual column distributions (77% versus 96%) and made preservation of pairwise correlations even more difficult (59% versus 95%).

### Overview of health data visualization tool

**Fig. 8:**
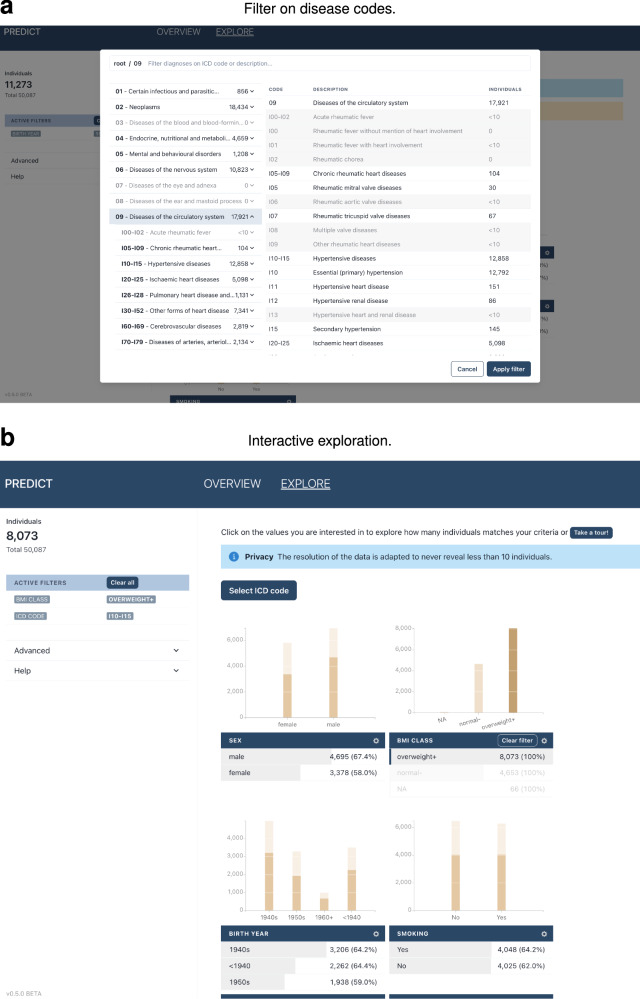
Interactive visualization tool for privacy-preserving exploration of biomedical data. **a** Filter on disease codes on different hierarchical levels in the ICD-10 classification, such as hypertensive diseases (ICD codes I10-I15). **b** Add more filters such as BMI class overweight or above to explore the number of individuals meeting certain criteria. The resolution of the data is adapted to never reveal less than 10 individuals.

We developed an interactive visualization tool that allows investigators to quickly explore the health data and assess study feasibility. Researchers could, for instance, examine whether sufficient numbers of individuals met specific criteria, such as the co-occurrence of lung cancer and obesity, and further stratify by sex or age distribution (see Fig. 8b). Beyond feasibility checks, the tool also supports hypothesis generation by enabling the exploration of unexpected correlations.

To prevent disclosure of sensitive information while reporting exact counts, we implemented an adaptive anonymization algorithm based on *k*-anonymity (see “Adaptive binning of sensitive data”).

The interface supports navigation through the hierarchical structure of ICD-10 disease codes (see Fig. 8a). For each code, including chapters and blocks, privacy-preserving datasets were generated and made available for exploration, provided they contained at least ten individuals.

### User guidelines and best practices

Based on our comprehensive evaluation of DGMs for biomedical data synthesis, several critical guidelines emerged for practitioners applying these methods in healthcare settings.

First, model choice was paramount. TabSyn variants consistently delivered the strongest results across statistical fidelity, ML utility, and DP metrics, making them the primary recommendation for biomedical synthetic data generation. In particular, TabSyn + CorrDst achieved average rankings around 3.4 across diverse datasets while maintaining computational efficiency. The correlation- and distribution-aware CorrDst loss not only improved preservation of feature dependencies but also enhanced training stability, and was therefore recommended over the vanilla loss function. When TabSyn was unavailable, CTGAN and TVAE could serve as alternatives, though they generally required larger sample sizes and longer runtimes to reach comparable quality.

Second, minimum data requirements demanded careful consideration. TabSyn reached stable performance with approximately 30,000 records, whereas traditional models often needed 40,000 to 60,000 samples. Paradoxically, Level 1 datasets with discrete variables required the largest sample sizes due to limited feature diversity and stricter DP constraints. Privacy-preserving synthesis under DP substantially increased computational burden, with models such as DP-CGANS and CTAB-GAN frequently encountering memory overflows, making them unsuitable for production applications.

Third, preprocessing proved critical for clinically meaningful outputs. Standard pipelines that replaced missing values with constants introduced severe correlation distortions and unrealistic distributions. The proposed structured pipeline combining median imputation, quantile transformations, and explicit missingness indicators consistently preserved distributional alignment and maintained essential clinical associations, particularly vital for high-missingness Level 3 datasets.

Finally, validation extended beyond technical metrics. Statistical similarity, ML utility, and privacy guarantees provided a strong quantitative foundation, but domain expertise remained indispensable. Human-in-the-loop validation was required to confirm biologically plausible relationships, such as sex-specific cancer incidence and links between smoking and lung cancer. Integrating expert review ensured that synthetic datasets met both quantitative benchmarks and clinical expectations.

Across all these dimensions, TabSyn + CorrDst paired with the proposed preprocessing pipeline consistently emerged as the most practical and reliable solution for generating high-fidelity, privacy-preserving synthetic datasets.

## Discussion

We present a comprehensive framework for health data anonymization that generates high-fidelity synthetic data while protecting patient privacy. The workflow includes data preprocessing, model training with hyperparameter optimization and a correlation- and distribution-aware loss function, systematic evaluation across multiple dimensions, and quality control validation. We provide the framework as open-source software with Docker containerization for deployment across diverse computing environments. Containerization eliminates technical barriers and ensures consistent execution across institutions, making state-of-the-art synthetic data generation accessible to the broader biomedical research community.

The framework introduces several methodological improvements for generating synthetic health data. Our preprocessing pipeline handles missing data through structured imputation, quantile transformations, and missingness indicators. This approach substantially improves on standard methods used in general-purpose synthetic data applications. Differential privacy (DP) evaluation ensures that generated datasets provide formal privacy guarantees while remaining useful for downstream analyses. Our ranking methodology aggregates performance across statistical fidelity, ML utility, and privacy metrics, offering objective guidance for model selection. Together with our previous work on correlation- and distribution-aware loss functions and iterative objective refinement Bayesian optimization (IORBO), these components establish a reliable and practical framework bridging theoretical advances in synthetic data generation with real-world biomedical requirements.

Our empirical evaluation identified several critical factors for successful generative modeling in biomedical contexts. Model selection strongly influenced outcomes. TabSyn variants consistently outperformed alternatives across statistical fidelity, ML utility, and DP metrics, making them our recommended choice for high-fidelity synthetic data generation. The correlation- and distribution-aware CorrDst loss improved feature dependency preservation and stabilized training. TabSyn + CorrDst achieved top-tier performance with moderate computational demands. CTGAN and TVAE provide viable alternatives to TabSyn, though they typically require larger datasets and longer runtimes for comparable results. Minimum data requirements varied by dataset complexity in a surprising way. Simpler Level 1 datasets with discrete variables often needed more records than more complex Level 2 or Level 3 cohorts due to limited feature diversity and stricter privacy constraints. Preprocessing proved essential. Conventional approaches that imputed missing values with constants distorted correlations and clinical patterns. In contrast, our proposed structured pipeline-combining median imputation, quantile transformations, and explicit missingness indicators-preserved realistic distributions and critical clinical relationships. This advantage was particularly evident in high-missingness Level 3 datasets. These findings reveal how model architecture, loss function design, dataset characteristics, and preprocessing combine in producing reliable, privacy-preserving synthetic health data.

The framework scales to clinical applications and applies broadly across sensitive healthcare datasets. We successfully evaluated it across vital statistics, cancer registries, and disease-specific cohorts of varying complexity, demonstrating that the system handles diverse biomedical data types. Runtime analyses show that leading models, particularly TabSyn and CTGAN, maintain feasible computational requirements for large-scale datasets, supporting practical institutional deployment. Quality control measures, including domain-specific sanity checks and expert validation, ensure that synthetic datasets preserve clinically meaningful relationships while meeting privacy requirements. Technical robustness combined with clinical validity positions the framework for use in epidemiological research, clinical trial design, drug discovery, and healthcare policy analysis, where data sharing constraints often limit collaboration.

Although the datasets used in this study vary substantially in structure, missingness patterns, and modeling tasks, they originate from a single national cohort ecosystem. Future work will therefore include validation on datasets from independent healthcare systems to further assess generalizability across heterogeneous data environments. Beyond healthcare cohorts, evaluating the framework on other sensitive tabular domains—such as financial records, mental health data, or governmental datasets—could further demonstrate its broader applicability to privacy-preserving data sharing in diverse settings.

The framework enables privacy-preserving data sharing while maintaining statistical fidelity, supporting collaborative research, regulatory compliance, and reproducibility in biomedical studies. Open-source software and containerized deployment ensure accessibility across institutions regardless of technical resources.

While external validation across independent healthcare systems remains an important next step, the technical barrier that has long limited practical privacy-preserving data synthesis in clinical research no longer stands. The proposed framework provides a scalable and reproducible approach to responsible data use, supporting data-driven biomedical research while maintaining public trust.

## Methods

### Implementation details

All DGMs evaluated in this study (CTGAN, CTAB-GAN, DP-CGANS, CopulaGAN, TVAE, TabDDPM, and TabSyn), along with the proposed correlation- and distribution-aware loss functions, were implemented using PyTorch version 1.13 (https://pytorch.org/). To ensure the reproducibility of our results, we maintained the original architectural designs and training protocols as specified in the respective publications. Additionally, to introduce variation in the comparison, we removed the conditional components from GAN-based models except for the CTAB-GAN. This was achieved by re-engineering the data sampling procedures to omit conditional vectors, thereby converting each model into its unconditional counterpart. All models were trained using the Adam optimizer^42^, with either default settings or parameters specified by the original authors.

Hyper-parameter tuning was conducted using the proposed IORBO framework, as detailed in ref. ^31^. This process was carried out in two distinct optimization stages, described in ref. ^31^, and aimed to ensure consistent performance across a range of datasets and model architectures.

For DP-enabled models (e.g., CTGAN, DP-CGANS and CopulaGAN), the formal privacy parameters *ϵ* and *δ* are incorporated as tunable hyper-parameters within the IORBO optimization. The optimization selects models that balance statistical fidelity, ML utility, and privacy, ensuring that the chosen models respect the computed privacy budget. Empirical post hoc DP metrics complement this formal accounting by providing practical validation of privacy protection in realistic scenarios.

All experiments were performed on a high-performance computing cluster equipped with NVIDIA A100 Tensor Core graphical processing units (GPUs) (each with 40GB of dedicated memory), Intel(R) Xeon(R) Gold 6338 CPUs, and 256GB of DDR4 RAM. Training durations varied significantly depending on the dataset and DGM complexity, ranging from a few hours up to 2 weeks.

To support efficient evaluation of downstream ML tasks, we utilized the cuML library^43^, which provided a GPU-accelerated Python API that closely mirrored the functionality of scikit-learn^44^. For consistency throughout the evaluation, we used scikit-learn to compute classification and regression metrics, scipy to derive statistical measures, and scikit-posthocs to perform statistical hypothesis testing.

For the first level (L1) and second level (L2) datasets, hyper-parameter tuning was carried out using the procedure described in “Relation to prior work”. In contrast, for the third level (L3) datasets, default hyper-parameter settings from the original implementations were used due to the substantial computational costs and GPU time requirements associated with tuning at this scale.

### Relation to prior work

Several components of our methodology were built directly upon and extend our earlier work^31^. The foundational loss functions introduced in that work were specifically designed to align the statistical properties of synthetic data with those of real datasets. By jointly preserving pairwise correlations and marginal distributions, the proposed loss functions improved the fidelity of synthetic tabular data across diverse feature types. In this work, we retained these core formulations while integrating them more broadly into a variety of DGMs. We further demonstrated their effectiveness across new benchmarks and in-house datasets.

#### Correlation- and distribution-aware loss functions

Let the training dataset be defined as:

$${\bf{X}}=\{{{\boldsymbol{x}}}_{i}=({{\boldsymbol{x}}}_{i}^{(c)},{{\boldsymbol{x}}}_{i}^{(d)}):\forall i\in \{1,\ldots ,N\}\},$$

where *N* is the number of training samples. Each sample ${{\boldsymbol{x}}}_{i}\in {{\mathbb{R}}}^{m}$ is composed of continuous features ${{\boldsymbol{x}}}_{i}^{(c)}$ and discrete features ${{\boldsymbol{x}}}_{i}^{(d)}$.

We adopted the loss functions introduced in our previous work^31^, which guide DGMs to match both pairwise feature correlations and marginal distributions of the target dataset. The *correlation-aware loss* was defined as:

1 $${{\mathcal{L}}}_{{\rm{cor.}}}=\frac{2}{m(m-1)}\mathop{\sum }\limits_{j=1}^{m}\mathop{\sum }\limits_{k=j+1}^{m}{({{\boldsymbol{g}}}_{j,k}-{\widetilde{{\boldsymbol{g}}}}_{j,k})}^{2},$$

where ***g***_*j*,*k*_ and ${\widetilde{{\boldsymbol{g}}}}_{j,k}$ denote the sample correlation between features *j* and *k* in the real and generated datasets, respectively.

To complement this, the *distribution-aware loss* was given by:

2 $${{\mathcal{L}}}_{{\rm{dis.}}}=\frac{1}{m}\mathop{\sum }\limits_{j=1}^{m}\mathop{\sum }\limits_{h=1}^{H}\frac{1}{h}{\left(1-\frac{{\widetilde{{\mathcal{S}}}}_{j}^{(h)}+\epsilon }{{{\mathcal{S}}}_{j}^{(h)}+\epsilon }\right)}^{2},$$

where ${{\mathcal{S}}}_{j}^{(h)}$ and ${\widetilde{{\mathcal{S}}}}_{j}^{(h)}$ denote the *h*-th order moments of feature *j* computed on the real and generated data, respectively. A small constant *ϵ* was added for numerical stability, and *H* denoted the number of moments considered.

These loss functions were used alongside standard DGM objectives to guide the training process. For complete derivations, theoretical analysis, and implementation details, we referred the reader to our previous study^31^.

In addition to the evaluated DGMs used in ref. ^31^, we included TabSyn^30^ in this work. For the TabSyn^30^, the proposed loss function was integrated into the total loss of the multinomial diffusions as:

3 $${\widetilde{{\mathcal{L}}}}_{{\rm{TabSyn}}}={\underbrace{{{\mathbb{E}}}_{{{\boldsymbol{z}}}_{0} \sim p({{\boldsymbol{z}}}_{0})}{{\mathbb{E}}}_{t \sim p(t)}{{\mathbb{E}}}_{{\boldsymbol{\varepsilon }} \sim {\mathcal{N}}({\boldsymbol{0}},{\boldsymbol{I}})}\parallel {{\boldsymbol{\epsilon }}}_{\theta }({{\boldsymbol{z}}}_{t},t)-{\boldsymbol{\varepsilon }}{\parallel }_{2}^{2}}}_{{{\mathcal{L}}}_{{\rm{TabSyn}}}}+\alpha {{\mathcal{L}}}_{{\rm{cor.}}}+\beta {{\mathcal{L}}}_{{\rm{dis.}}},$$

where ${{\mathcal{L}}}_{{\rm{TabSyn}}}$ denotes the original TabSyn achieved via denoising score matching^45^.

#### Hyper-parameter optimization

We continued to employ the IORBO framework^31^ for hyper-parameter tuning, which integrated Bayesian optimization with iterative target refinement to effectively explore complex, non-convex objective landscapes. Specifically, we leveraged the tree-structured Parzen estimator approach (TPE) algorithm^46^ implemented in the Hyperopt (https://hyperopt.github.io/hyperopt/) library. As in our previous work, we conducted two distinct tuning processes: (1) for downstream ML tasks, we fine-tuned each model using five-fold cross-validation on task-specific evaluation metrics, and (2) for DGM, we optimized hyperparameters for each generator-dataset-loss combination. In this work, we extended the optimization objective to include additional regularization terms and downstream utility metrics, improving the flexibility and robustness of the tuning process.

#### Statistical tests

In our statistical analysis, we followed standard practices for hypothesis testing as outlined in ref. ^31^. Specifically, we interpreted *p* values using common thresholds for significance: *p* ≤ 0.01 indicated highly significant results, 0.01 < *p* ≤ 0.05 indicated moderate significance, and *p* > 0.05 suggested insufficient evidence to reject the null hypothesis. For comparing loss functions across generative models and datasets, we employed the Friedman test^47^—a non-parametric alternative to repeated-measures ANOVA—for ranking the methods based on two key metrics: test set predictions and statistical similarity between real and synthetic data. To identify significant differences between the ranked methods, we performed post-hoc pairwise comparisons using the Nemenyi test^48^. The results of these tests were summarized in ref. ^31^, where *p* values corresponded to both positive and negative differences across methods.

#### Evaluation

Our evaluation protocol (see Supplementary Material Fig. S4a) was built upon the comprehensive framework introduced in ref. ^31^, where we quantified the statistical similarity between real and synthetic datasets using distributional and correlation-based measures. Consistent with this prior work, we again applied non-parametric hypothesis tests (e.g., KS, Chi-squared) and effect size measures to assess differences in individual variable distributions and pairwise dependencies. We further evaluated ML performance on both Train-Synthetic-Test-Real (TSTR) and augmentation tasks, using a broad suite of classification and regression metrics under cross-validation.

In this work, we extended the framework in two directions to improve practical utility and robustness. First, we integrated DP risk assessments to quantify the re-identification risk of synthetic samples, offering a privacy-preserving perspective. Second, we explicitly evaluated ML performance under class imbalance, using metrics such as balanced accuracy, G-mean, and AUC to assess robustness in skewed-label scenarios. These additions, detailed in Imbalance-Aware Learning and Evaluation-3 and Supplementary Material Table S4a, supported a more realistic evaluation of synthetic data in privacy-sensitive and imbalanced-data contexts.

#### Benchmarking framework

Finally, the benchmarking framework used to assess model utility was adapted from ref. ^31^, which provided a standardized framework for evaluating DGMs across classification, regression, and statistical similarity tasks. In the present work, we expanded this framework to handle imbalanced datasets and privacy-aware applications (see Fig. S4 in the Supplementary Material), ensuring that both utility and privacy were jointly assessed. Together, these refinements represented a natural evolution of our earlier contributions, broadening their scope and strengthening their relevance to privacy-preserving data synthesis.

### Dataset generation

Datasets are generated from sensitive biobank data through a three-level process that progressively transforms raw health records into analysis-ready representations (see Fig. 1). The pipeline begins with data cleaning and harmonization, followed by structured transformation and refinement for downstream use. These stages are denoted as Level 1, Level 2, and Level 3.

#### First level

The generation of the first level biomedical datasets involved a systematic transformation of raw data from the PREDICT, cancer registry, and consent records to create a unified, anonymized dataset suitable for subsequent analysis (see Fig. 1). The process commenced with the integration of three distinct tabular datasets into a single framework through an outer merge based on a unique patient identifier, ensuring comprehensive representation of patient information. To enhance relevance, the dataset was refined to include only individuals recruited in the PREDICT cohort, identified by a specific cohort indicator. Temporal attributes, such as birth, sampling, questionnaire, death, and vital status dates, were converted into life span metrics by calculating year differences, with age determined as the maximum available span.

Subsequent steps focused on structuring and anonymizing the data to safeguard sensitive information while preserving analytical value. Dates were grouped into 5-year intervals to reduce granularity and protect individual identities, while life span metrics underwent similar categorization to support aggregated analysis. To mitigate sparsity and ensure statistical robustness, records with infrequent occurrences of categorical variables-such as geographic locations and diagnostic codes-were excluded if their unique patient identifier counts were below a threshold of 5. Sensitive fields, including diagnostic classifications and project identifiers, were pseudonymized by encoding categorical values into numerical representations, and the patient identifier was randomized, renumbered, and reformatted to further enhance anonymity. Finally, continuous variables like body mass index were rounded for consistency, and extraneous or sensitive attributes were removed, resulting in a refined dataset stored for use in downstream modeling endeavors. Algorithm 1 details the data generation procedure for first-level datasets.

##### Algorithm 1

Data generation for first-level datasets

1:**Input:** Raw datasets, patient ID, cohort ID

2: **Output:** Processed dataset

3: Merge datasets using patient ID

4: Filter for PREDICT cohort

5: Calculate life spans and age

6: Bin dates and spans into 5-year groups

7: Remove rare categories

8: Anonymize sensitive data

9: Clean and round data

10: **Return** Processed dataset

#### Second level

The generation of the second-level biomedical datasets involved a detailed integration and refinement of data from questionnaire, registry, journal, and metabolomics sources to construct a comprehensive dataset tailored for cancer-related analyses (see Fig. 1). The process began by importing four distinct datasets-questionnaire responses, International Classification of Diseases (ICD) registry records, patient journals, and metabolomics profiles-into a unified framework, with initial cleaning steps such as removing redundant columns like cohort identifiers and event types from the questionnaire data. These datasets, representing 50,087 unique patient identifiers from questionnaires, 14,698 from registries, 32,996 from journals, and 3284 from metabolomics, were merged sequentially, starting with a left join of questionnaire and registry data based on a common patient identifier, followed by an inner join with metabolomics data using a sample identifier linkage.

A key aspect of the preprocessing was the classification and aggregation of cancer-related information to enhance analytical specificity. Diagnostic codes from ICD7 and ICD9 systems were labeled with specific cancer types—such as prostate, breast, colorectal, urothelial and kidney, lung, pancreatic, and hematological. We used predefined code ranges and prefixes, with a final cancer column created by prioritizing ICD9 labels where available. To capture temporal variations in patient measurements, a temporal data consolidation function restructured event-based data (e.g., “exclude diet”, “height”, “total cholesterol”) into up to five chronological sets per patient, filling earlier sets with missing values where data was sparse, particularly evident in the high missingness rates (e.g., 99.92% for “exclude diet” second set).

The final preprocessing stages focused on data reduction and quality enhancement to prepare the dataset for modeling. Columns with high missingness, such as initial measurement sets (e.g., “exclude diet” or “event date”) and metabolomics variables (e.g., EDTA plasma at 91.33%), were dropped to mitigate noise, while retaining critical attributes like birthdate and death date. Text data were standardized by stripping whitespace and converting to lowercase, and the resulting merged dataset was saved for further use. Despite significant missingness in later measurement sets (e.g., 41.85% for “event date”) and cancer-related fields (e.g., 64.50% for “diagnosis date”), the process generated cancer-specific subsets (including Prostate, Breast, Colorectal, Urothelial and Kidney, Lung, Pancreatic, and Hematological), enabling targeted investigations while addressing data sparsity through strategic filtering and aggregation. Algorithm 2 details the data generation procedure for second-level datasets.

##### Algorithm 2

Data generation for first-level datasets

1: **Input:** Raw datasets (questionnaire, cancer registry, journals, metabolomics)

2: **Output:** Processed dataset

3: Aggregate questionnaire events by patient

4: Label cancer types using ICD-7 and ICD-9 codes

5: Merge datasets using patient ID

6: Combine cancer labels into a single column

7: Remove sensitive and redundant columns

8: Clean and standardize data

9: Save processed dataset

10: **Return** Processed dataset

#### Third level

The generation of the third-level biomedical datasets followed the same overall procedure as the Second Level but incorporated more complete data sources. To simplify disease-specific datasets, we used the ICD-9 codes truncated to three-character resolution and translated from ICD-7 where ICD-9 entries were missing. The datasets were aggregated on a per-patient basis using the first available records, after which sensitive and redundant columns were removed. The questionnaire data were exported and merged with a multiclass cancer variable based on each patient’s first available ICD-9 code, when present.

For each ICD-9 diagnosis with sufficient data, we additionally exported questionnaire datasets merged with a binary cancer indicator (set to true if the patient had ever received the corresponding diagnosis). The most prevalent diagnoses were prostate cancer (ICD-9 code 185; 2962 patients), breast cancer (code 174; 1971 patients), and colon cancer (code 153; 1077 patients). Furthermore, we exported the aggregated questionnaire data combined with the complete metabolomics collection.

In total, 11 datasets were generated under final general data protection regulation (GDPR)-compliant approval from a special regional ethical review board. Algorithm 3 summarizes the data generation procedure for the third-level datasets.

##### Algorithm 3

Data generation for third-level datasets

1: **Input:** Raw datasets (questionnaire, cancer registry, journals, metabolomics)

2: **Output:** Processed dataset

3: Remove rows with missing ICD codes or malignancy status benign

4: Trim ICD codes to three letters

5: Translate ICD-7 to ICD-9 for rows with missing ICD-9 codes

6: Aggregate questionnaire data by patient using the first available records

7: Remove sensitive and redundant columns

8: Clean and standardize data

9: **for** each unique ICD-9 code **do**

10:  Add a Boolean cancer column to the questionnaire data and export

11: **end** **for**

12: Add a multiclass cancer column to the questionnaire data and export

13: Merge with metabolomics data and export

14: **Return** Processed datasets

### Preprocessing pipeline for model training

Preparing high-missingness biomedical data for generative modeling requires three linked decisions: how to impute missing values, how to stabilize heavy-tailed distributions, and how to represent missingness explicitly rather than removing it. To address these challenges, we design preprocessing strategies tailored to different dataset levels, ensuring compatibility with DGMs while preserving data quality and clinically meaningful structure.

#### First and second level

For Level 1 and Level 2 datasets, missing values in both continuous and discrete variables were imputed with a constant value of −1, while categorical variables were encoded using one-hot representations. This preprocessing strategy had been shown to perform adequately in natural, non-sensitive datasets where the extent of missingness was limited^31^.

#### Third level

For the third-level datasets, which contained substantially higher levels of missing data, a structured preprocessing pipeline was implemented to systematically address missingness while preserving data integrity for downstream analysis. The pipeline began with the encoding of *date-related features*, converting them into numerical representations of days since a fixed reference point. This ensured consistency across records and facilitated the treatment of missing or malformed entries.

Continuous and date variables with missing values were imputed using the median of the observed distribution, after which a quantile transformation was applied. Unlike standard normalization, which only rescales values linearly, quantile transformation maps the data to a uniform or Gaussian distribution, reduces the impact of outliers, and produces more stable feature distributions for training DGMs.

Binary and categorical variables were processed using one-hot encoding, ensuring that each category was represented as an independent dimension. This approach avoids implicit ordinal relationships while maintaining compatibility with downstream learning algorithms.

To further enhance model interpretability, the pipeline generated indicator variables for each continuous feature to flag instances of missingness. This allows learning algorithms to capture informative patterns associated with missing data rather than discarding them. Discrete variables are retained in their original form where appropriate to ensure structural fidelity and enable accurate inverse transformations.

In the third-level datasets, only 28 of the 126 variables were continuous, meaning that the introduction of missingness indicators resulted in a relatively small increase in dimensionality. Moreover, the inclusion of these indicators does not affect the differential privacy budget, as the privacy parameter *ϵ* in standard differentially private training mechanisms depends on factors such as sampling rate, training iterations, and noise multiplier rather than the number of input features. In datasets with substantially larger numbers of continuous variables, indicators can also be selectively introduced only for variables with high missingness (e.g., ≥50%), which further limits dimensional growth.

Finally, the preprocessing pipeline was designed to be bidirectional, supporting reconstruction of the original data format, including data types. In particular, the quantile transformation used in the pipeline is deterministic and strictly monotonic, constructed from the empirical cumulative distribution function of each variable. When the same fitted transformer is used for inverse transformation, the mapping remains bijective within the empirical quantile grid, ensuring that each transformed value corresponds to a unique value on the original scale. This property preserves the rank ordering and distributional structure of the data during both forward and inverse transformations. This design was essential for interpretability, validation, and reproducibility of analyses. Collectively, these steps mitigated the effects of high missingness while ensuring that the datasets remained suitable for both statistical modeling and ML applications.

##### Algorithm 4

Preprocessing pipeline for third-level biomedical datasets

1: **Input:** Raw dataset with continuous, date, binary, and categorical variables

2: **Output:** Preprocessed dataset with bidirectional transformations

3: Encode date features as number of days since fixed reference point

4: **for** each continuous and date variable **do**

5:  Impute missing values with median of observed distribution

6:  Apply quantile transformation to stabilize distributions and reduce outlier effects

7:  Generate missingness indicator to flag originally missing values

8: **end** **for**

9: **for** each binary and categorical variable **do**

10:  Replace missing values with dedicated placeholder

11:  Apply one-hot encoding to avoid implicit ordinal relationships

12: **end** **for**

13: Retain discrete variables in original form to preserve structural fidelity

14: Ensure bidirectional transformation for full reconstruction of original data types

15: **Return** Preprocessed dataset ready for modeling

#### Illustrative example

To demonstrate the preprocessing pipeline in the Third Level, we consider a fictive dataset with five patients shown in Table 1. The dataset includes a date variable (diagnosis_date), one continuous feature (blood_pressure), and one categorical feature (smoking_status). Several values are missing, indicated by *n.a*.

**Table 1:** Raw input dataset (fictive example)

ID	diagnosis_date	blood_pressure	smoking_status
1	2010-05-20	120	Former
2	*n.a*.	*n.a*.	Current
3	2015-07-13	180	*n.a*.
4	2003-02-01	95	Never
5	*n.a*.	140	Current

The preprocessing steps were applied as follows:

1. Date encoding: convert diagnosis_date into the number of days since a fixed reference (e.g., 2000-01-01).
2. Imputation and quantile transform (continuous and date): impute missing values using the median of observed entries, then apply a quantile transformation. Unlike standard normalization, quantile transformation reshapes skewed or heavy-tailed variables toward a uniform or Gaussian distribution, thereby reducing the impact of outliers and yielding more stable inputs for generative modeling.
3. Missingness indicators: add binary flags to record whether a value was originally missing prior to imputation (e.g., diagnosis_q_m, bp_q_m).
4. Categoricals: replace missing categories with a placeholder ("Unknown”) and apply one-hot encoding.

The resulting processed dataset is presented in Table 2. Quantile-transformed values were scaled between 0 and 1 for illustrative purposes.

**Table 2:** Processed dataset (fictive example)

ID	diagnosis_q	bp_q	diagnosis_q_m	bp_q_m	smoking_status (one-hot)			
					Current	Former	Never	Unknown
1	0.50	0.35	0	0	0	1	0	0
2	0.50	0.50	1	1	1	0	0	0
3	0.95	0.95	0	0	0	0	0	1
4	0.05	0.10	0	0	0	0	1	0
5	0.50	0.65	1	0	1	0	0	0

Quantile values are illustrative (0–1). (i) diagnosis_q and bp_q represent the quantile-transformed versions of the encoded date and blood pressure after median imputation. (ii) diagnosis_miss and bp_miss indicate whether the original value was missing (1) or observed (0). These features allowed the model to exploit informative patterns of missingness. (iii) One-hot encoding avoided introducing spurious ordinality into smoking_status, with the “Unknown” category explicitly capturing originally missing values.

### Adaptive binning of sensitive data

To reveal the exact number of individuals meeting certain criteria without revealing sensitive information, we developed an adaptive anonymization algorithm based on *k*-anonymity^49^. The goal was to limit re-identification risk by ensuring that for any combination of quasi-identifiers (attributes like zip code, age, and sex), there were at least *k* individuals with those same attributes, preventing attackers from pinpointing a unique person. In standard *k*-anonymity, a dataset typically contained sensitive attributes such as diagnosis and income that should not be linkable to a specific person. These were kept, but protected indirectly by anonymizing the quasi-identifiers, commonly by suppression (removing values) or generalization (coarse-graining, for example replacing age with age groups).

While *k*-anonymity safeguards against identity revelations, it does not necessarily protect against the disclosure of specific attributes. This becomes problematic when attackers possessed background knowledge that could be combined with quasi-identifiers to reduce the set of possible values for the sensitive attribute. Additionally, the absence of diversity in sensitive domains could result in the exposure of personal information through homogeneity attacks, for example if all *k* people in a group have the same disease. An extension called *l*-diversity was proposed to address this by ensuring that there were at least *l* well-represented values for the sensitive attribute. However, the sensitive values can be skewed or semantically similar and therefore also revealing, for example all diseases belonging to the stomach or all income values being low. A further extension called *t*-closeness was introduced to address this, ensuring that the distance between the distribution of a sensitive attribute for each equivalence class (any combination of quasi-identifiers) and the distribution of the attribute in the whole dataset was no more than a threshold *t*^50^.

Our solution to these vulnerabilities recognized that the root of them was the split of attributes as either identifying or non-identifying when in fact all attributes were potentially identifying, depending on their prevalence in the population and on auxiliary data that the attacker might have. By treating all attributes as both quasi-identifiers and potentially sensitive, we avoided the potential vulnerabilities that *l*-diversity and *t*-closeness tried to solve, and by using a fast greedy search with fallback to an exhaustive brute force check, we ensured that no combination of filters on any column gave less than *k* individuals, thus protecting against both homogeneity attacks and background knowledge attacks.

The trade-off between privacy and utility was the same as for *k*-anonymity, where the precision or number of values for each attribute quickly dropped with the number of attributes. To keep utility, we selected a few common attributes to get an overview: birth year, BMI, sex, and smoking status.

For each non-binary attribute, we constructed bins with different levels of precision, for example birth year binned to 5-year bins, then 10-year bins, and so on. For each level, we aimed to divide the data into bins so that each bin contained roughly the same number of observations. However, the value of equal-frequency binning for optimized precision had to be balanced with the value of simple and meaningful intervals. For example, our first-level bins on birth year were decades, with the end bins adapted to include the tails with enough data points, in our case less than 1940, 1940s, 1950s, and 1960+. Our second level of bins was less than 1950 and 1950+. For BMI, we used clinically defined bins for underweight (less than 18.5), normal (18.5 to less than 25), overweight (25 to less than 30), and obesity (30 or greater). As a next level, we merged two categories at each end into normal- and overweight+.

To adapt the bins to fulfill privacy requirements, we used *k* = 10 for total *k*-anonymity, that is, *k*-anonymity assuming all attributes were quasi-identifiers, and iteratively coarse-grained or removed an attribute until that was fulfilled.

#### Algorithm 5

Adaptive binning for privacy protection

**Require**: Dataset *D*, minimum group size *k*, ordered candidate binners ${\mathcal{B}}$

**Ensure**: Privacy-preserving dataset *D*_bin_

 1: *D*_bin_ ← apply first binner from ${\mathcal{B}}$ to each binned column

 2: **while** **not** PrivacySatisfied(*D*_bin_, *k*) **do**

 3:   (c^*^, v^*^, n^*^) ← GreedyFilter(*D*_bin_)

 4:   **if** coarser binning exists for column c^*^ **then**

 5:    apply next coarser binner to column c^*^

 6:   **else**

 7:    remove column c^*^ from *D*_bin_

 8:   **end if**

 9: **end while**

10: **return** *D*_bin_

### Imbalance-aware learning and evaluation

To improve the robustness and fairness of model evaluation in imbalanced classification settings, we introduced several modifications to the previous implementation^31^. First, we applied class weighting to account for the unequal distribution of classes during model training. For both support vector machine (SVM) and logistic regression classifiers, we specified the parameter class_weight = "balanced" to ensure that each class contributed proportionally to the loss, thus mitigating bias toward majority classes.

In addition, for models that support sample-specific weights (such as XGBoost), we provided a custom weight vector sample_weights_xgb_multi when available. This allowed the model to explicitly compensate for class imbalances during optimization. If no such weight vector was present, the model defaulted to standard fitting without sample weighting.

To evaluate performance under class imbalance, we adopted metrics that were sensitive to skewed class distributions. Specifically, we reported the weighted versions of precision, recall, and *F*1-score-each of which considered per-class performance while adjusting for class frequency. We also computed the geometric mean score (G-mean), which captured the balance between sensitivity (recall) and specificity across all classes, making it particularly effective in the presence of imbalanced data. By integrating both training-level adjustments and tailored evaluation metrics, our framework ensured a more realistic and comprehensive assessment of synthetic data utility in real-world classification problems.

### Differential privacy evaluation

To assess the privacy risks of synthetic datasets, we evaluated several DP-inspired metrics that simulate different attack scenarios. The main goal was to quantify the extent to which real data records could be inferred, re-identified, or linked from the synthetic data.

#### Algorithm 6

Privacy verification

 1: **function** PrivacySatisfied(*D*, *k*)

 2:   *ok* ← GreedyPrivacyTest(*D*, *k*)

 3:   **if** *ok* **then**

 4:    *ok* ← BruteForcePrivacyTest(*D*, *k*)

 5:   **end if**

 6:   **return** *ok*

 7: **end function**

 8: **function** GreedyPrivacyTest(*D*, *k*)

 9:   **while** columns remain in *D* **do**

10:    (c^*^, v^*^, n^*^) ← GreedyFilter(*D*)

11:    **if** $${c}^{* }=\varnothing$$ **then**

12:     **return** **true**

13:    **end if**

14:    **if** n^*^ < *k* **then**

15:     **return** **false**

16:    **end if**

17:    *D* ← {*r* ∈ *D*∣*r*[c^*^] = v^*^}

18:    **end while**

19:    **return** **true**

20: **end function**

21: **function** BruteForcePrivacyTest(*D*, *k*)

22:   **if** no columns remain **then**

23:    **return** **true**

24:   **end if**

25:   **for** each column *c* in *D* **do**

26:    **for** each value *v* appearing in column *c* **do**

27:     $${D}^{{\prime} }\leftarrow \{r\in D| r[c]=v\}$$

28:     $$n\leftarrow | {D}^{{\prime} }|$$

29:     **if** *n* < *k* **then**

30:      **return** **false**

31:     **end if**

32:     remove column *c* from ${D}^{{\prime} }$

33:     **if not** BruteForcePrivacyTest(${D}^{{\prime} },k$) **then**

34:      **return** **false**

35:     **end if**

36:    **end for**

37:   **end for**

38:   **return** **true**

39: **end function**

40: **function** GreedyFilter(*D*)

41:   $${c}^{* }\leftarrow \varnothing$$

42:   n^*^ ← ∣*D*∣

43:   **for** each column *c* in *D* **do**

44:    find value *v* with smallest frequency *n*

45:    **if** *n* < n^*^ **then**

46:     (c^*^, v^*^, n^*^) ← (*c*, *v*, *n*)

47:    **end if**

48:   **end for**

49:   **return** (c^*^, v^*^, n^*^)

50: **end function**

## *k*-Anonymity

We computed *k*-anonymity by clustering the data using K-Means over a set of quasi-identifying key features. The minimum number of records sharing the same cluster across different values of *k* (e.g., 2, 5, 10, 15) determined the effective *k*-anonymity score. Higher values indicated stronger privacy guarantees. We applied this method separately to both real and synthetic datasets and compared their anonymization levels.

## *l*-Diversity

To capture intra-cluster attribute variability, we extended the previous approach to compute distinct *l*-diversity. For each cluster, we assessed the number of unique sensitive attribute combinations. The minimum distinct count across clusters was reported as the *l*-diversity score. A higher score implied better resistance to attribute inference attacks.

## *k*-Map

Unlike *k*-anonymity, which evaluates identity indistinguishability within the dataset, *k*-map considers re-identification risk under the assumption that an adversary has access to external data. We fit K-Means to the real data and measured how well synthetic samples were distributed across the real data’s clusters. The smallest cluster count (i.e., the most vulnerable group) informed the final *k*-map score.

## Δ-Presence

Δ-presence estimates the probability that an individual from the real dataset is also present in the synthetic dataset. By comparing the overlap of sensitive attributes between corresponding clusters in the real and synthetic datasets, we computed the maximum presence ratio, which served as an upper bound on re-identification risk. Lower Δ-presence values are preferred.

Each metric was computed using multiple clustering granularities to account for structure at different resolutions. We also implemented fallback logic to avoid unreliable results when sample sizes per cluster were insufficient. These privacy metrics provide complementary insights into the disclosure risks of synthetic datasets, making them an essential component of our expanded evaluation framework.

## Re-identification score

The Identifiability Score from ADS-GAN^51^ estimates the proportion of real data points that are closer to synthetic samples than to their second nearest real neighbor. We implemented this metric to quantify privacy; a lower score indicates better privacy.

## Membership inference attacks (MIA)

Membership inference attacks evaluate the likelihood that a classifier can determine whether a record was part of the training data. We implemented a density-based inference method inspired by DoMIA^52^, measuring both MIA accuracy and MIA area under the ROC curve (AUC). Lower values imply higher resistance to membership inference.

## Correct attribution probability (CAP)

We included two variants of CAP^37^:

- Zero CAP assumes an attacker has exact knowledge of key attributes and tries to guess a sensitive attribute.
- Generalized CAP (Gen. CAP) assumes only approximate attacker knowledge.

Higher values indicated more uncertainty for the attacker, hence better privacy.

## Single-out score

The single-out score estimates the probability of singling out a real individual from the synthetic data. It is calculated as the chance of any real record being closer to a synthetic one than its second closest real neighbor^51^. Lower scores indicate better privacy. We applied this metric to assess disclosure risk.

## Linkability score

The linkability score measures the ability to link different synthetic records back to a unique real individual, based on proximity in feature space. It reflects structural overlap between real and synthetic distributions. We computed this metric across datasets to quantify potential linkage attacks.

## Inference risk

Inference risk evaluates the probability of inferring hidden or sensitive attributes given partial information. CAP-based measures form the basis of this type of privacy evaluation. We calculated inference risk for each dataset.

These metrics provide a multifaceted perspective on privacy, allowing us to quantify attack risks in both worst-case and probabilistic scenarios. We implemented these metrics based on or adapted from open-source privacy evaluation libraries such as SynthCity^36^ and SDMetrics^37^.

## Evaluation and ranking of generative models

In this study, we employed a rigorous methodology to evaluate and rank DGMs for synthetic data generation within biomedical contexts. The process commenced with the aggregation of performance metrics from all evaluated DGMs, including CTGAN, CopulaGAN, DPCGANs, CTAB, TVAE, TabDDPM, and TabSyn, across an extensive collection of health-related datasets. These metrics, derived from hyper-parameter optimization trials, were systematically compiled into a structured dataset, ensuring that each model’s optimal configuration was represented. This comprehensive dataset, spanning various biomedical scenarios, provided a foundation for comparative analysis, enabling a thorough assessment of model efficacy across different applications.

The ranking procedure relied on the Nemenyi test, a well-established statistical method for post-hoc comparisons, to discern significant differences in model performance across multiple datasets and evaluation metrics. Performance scores were calculated for each model, followed by the assignment of ranks based on average metric outcomes, with appropriate handling of tied results. The test determined a critical difference (CD) value at a 95% confidence level (*p* = 0.05), facilitating the identification of statistically significant performance variations.

The resulting rankings were organized into a structured format, mapping each dataset to a corresponding set of model rankings. This approach ensures that the identified top-performing models, excelling in aspects including statistical fidelity, ML utility, DP, or comprehensive performance (encompassing all evaluated metrics), were statistically validated and relevant to biomedical applications. Consequently, this approach provides a reliable basis for selecting the most effective DGM for synthetic data generation.

## Postprocessing and rejection sampling

Postprocessing is a critical step in synthetic data generation, addressing the common issue that DGMs, while capable of capturing overall data distributions, often failed to preserve intricate inter-variable relationships. In particular, high-dimensional biomedical datasets frequently contain complex correlations that were essential for downstream analyses but were not always fully respected. To mitigate these discrepancies, we employed *rejection sampling*, a post-hoc strategy that selectively accepted or discarded generated samples based on pre-defined statistical or logical criteria. Each synthetic sample was evaluated against the target distributions and correlation structures derived from the original data, and only those meeting the acceptance criteria were retained. This process ensured that the resulting synthetic dataset more faithfully replicated key statistical properties, including correlations, distributions, and conditional dependencies, without modifying the underlying DGM.

In our framework, postprocessing was guided by domain-specific rules defined by human experts as part of the *Quality Control Measures*. For example, certain malignancies were inherently sex-specific: cervical, placental, endometrial, ovarian, and other female genital cancers were observed only in females, whereas prostate, testicular, and other male genital cancers occurred exclusively in males. Similarly, well-established clinical relationships, such as the strong correlation between lung cancer and smoking status, or between body mass index and weight, had to be preserved. These rules, formulated by domain experts, served as explicit constraints during postprocessing, ensuring that the synthetic data respected both biological plausibility and clinical logic. By embedding such expert-driven criteria, we corrected unrealistic or invalid synthetic samples that would otherwise compromise the fidelity and utility of the dataset.

In practice, we applied postprocessing and rejection sampling to the synthetic datasets generated at the last stage of the pipeline. After initial generation, a candidate batch of synthetic samples was produced, and each sample was evaluated against both statistical criteria (e.g., correlation distances, distributional alignment) and expert-defined logical rules. Samples failing to meet these criteria were discarded, and additional samples were generated iteratively until the desired dataset size and quality was achieved. This approach allowed us to produce high-fidelity synthetic data that maintained key statistical relationships, adhered to clinical constraints, and satisfied privacy requirements. Combined with automated quality control and expert review, postprocessing ensured that the final synthetic datasets were both analytically reliable and biologically plausible, enabling downstream analyses and privacy-preserving data sharing in biomedical research.

For DP-enabled models (e.g., DP-CGANS), the rejection sampling step does not access private training data and therefore does not alter the formal DP guarantees established during model training, whereas for non-DP models, postprocessing purely enhances clinical plausibility without formal privacy protection.

## Data sufficient analysis

In this study, we performed a comprehensive data sufficiency analysis to identify the minimum number of samples required to effectively train DGMs capable of producing high-fidelity synthetic data in biomedical contexts (see Fig. S4b in the Supplementary Material). We employed a systematic sampling strategy, drawing subsets of increasing sizes–starting from 1000 and incrementally increasing through 1500, 2000, up to 70,000 samples–from the original dataset. This design allowed us to evaluate model performance across a broad spectrum of training data volumes. For each sampled dataset, we performed hyper-parameter tuning using IORBO to maximize performance. The best-performing model for each sample size was then selected based on this tuning procedure.

Following the identification of the optimal model for each sample size, we evaluated each optimal model using benchmarking framework including statistical fidelity, ML utility, and DP. Each optimal model was then used to generate synthetic datasets approximately equal in size to their corresponding training subsets, enabling direct comparisons between real and synthetic data across sampled sizes.

The final stage of this methodology involved a rigorous synthetic evaluation process to assess the quality of the generated data (see Supplementary Material Fig. S4). This evaluation was conducted twice: initially on the synthetic data produced by each optimal model, and subsequently on a refined synthetic dataset generated by the best-performing model identified through IORBO. The evaluation incorporated the same suite of metrics—statistical, ML, and DP—to ensure consistency and robustness. By iterating through the “Test” phase, the sufficiency of the training data was validated, allowing us to pinpoint the minimum sample size necessary to achieve “good” synthetic data, defined by high performance across all evaluated metrics. This iterative and data-driven approach provided a reliable foundation for determining the optimal data requirements for effective generative modeling in biomedical applications.

To determine the threshold for data sufficiency, a ranking-based method was employed. This approach aggregates performance data across multiple biomedical datasets, including patient and record data, for various DGMs including CTGAN, TVAE, and TabSyn. For each dataset and model configuration, the proposed method preprocesses the data by filtering inconsistent metrics and applying the Nemenyi test to compute ranks based on average performance scores across different sample sizes. The elbow point, identified through a smoothing spline and KneeLocator algorithm^41^ applied to these ranks, indicates the minimum sample size where performance stabilizes, offering a statistically robust threshold that reflects significant performance differences.

Alternatively, a composite score-based method was utilized to establish data sufficiency thresholds. This method consolidates individual metric scores-spanning statistical, ML, and DP evaluations-into a unified composite score for each sample size. This composite score is computed as the mean score across metrics after normalization within predefined threshold ranges. By applying a smoothing spline and KneeLocator^41^ to this composite score curve, the method identifies the elbow point, representing the minimum sample size where the score plateaus. This approach, visualized through plots, provides an intuitive and comprehensive assessment of data sufficiency, integrating diverse performance aspects into a single threshold estimate.

## Quality control implementation details

### Utility

To assess the clinical plausibility and practical utility of the synthetic data, we conducted a series of sanity checks informed by established medical knowledge. The goal was to assess whether medically expected associations-well-documented in epidemiological literature, and verifiably present in a reference dataset from the original health data-were preserved in the synthetic outputs.

We focused on basic anthropometric relationships and associations between specific cancers and known risk factors, such as smoking and lung cancer. Particular attention was paid to sex-specific associations, such as the exclusive occurrence of prostate cancer in males and the marked predominance of breast cancer in females. Deviations from these patterns were flagged as biologically implausible and indicative of model failure.

These expected associations were assessed using frequency tables and logistic regression models, independently of the core model development process. The sanity checks served as an iterative feedback mechanism, identifying biologically implausible patterns that prompted targeted adjustments to model parameters and input representations. This domain-driven validation complemented technical metrics and helped ensure that the synthetic output more closely reflects real-world clinical data. This process highlights the critical role of domain expertise in identifying clinically relevant inconsistencies that may be overlooked by purely statistical validation.

To further enforce these domain-driven constraints, the correlations and logical rules identified by experts during quality control are incorporated into a postprocessing step using rejection sampling (see “Postprocessing and rejection sampling”). Specifically, the expert-defined associations-such as sex-specific cancers or clinically established correlations like lung cancer and smoking-serve as explicit criteria to evaluate each synthetic sample. Samples that violate these rules are rejected, and additional data are generated iteratively until the synthetic dataset satisfies both statistical and domain-specific requirements. This integration ensures that the final synthetic outputs not only meet quantitative benchmarks but also maintain clinical plausibility, bridging technical validation with expert-informed domain knowledge.

To validate the statistical similarity between synthetic and real data, we generated a Quality Report and Diagnostic Report using the Synthetic Data Metrics (SDMetrics) library^37^. The results across the biomedical datasets are summarized in Table S3 in the Supplementary Material. The Quality Score is a combination of Column Shapes and Column Pair Trends. The first measures the average statistical similarity of the synthetic and real distributions of each column (see Fig. 7b). The second scores how well the synthetic data capture the pairwise correlations of the real data. The Diagnostic Score, expected to be 100% or very close, is a combination of Data Validity and Data Structure. The first verifies that the synthetic data are within the correct ranges (or category options), and the second verifies that the shape and column names are the same.

### Privacy

We evaluated privacy risks using complementary approaches. The first of which was a pragmatic, heuristic approach that involved assessing how closely synthetic records resemble real ones, using four sets of pseudo-identifiable variables. Special attention was given to the impact of including sensitive cancer diagnostics data. Matching was performed using a configurable logic that allowed for approximate matches within predefined spans for each variable.

To establish a baseline for natural similarity in the dataset, we repeated the same matching procedure within the real data itself. In this comparison, each real observation was matched against all other real observations excluding those from the same individual, thereby simulating the likelihood of coincidental matches between different individuals.

For each configuration (1–4), we calculated the proportion of synthetic observations that matched at least one real observation on all included variables, and compared this to the match rate observed in the real-to-real baseline.

As shown in Fig. 7, synthetic data consistently exhibited lower match rates than real data, supporting its privacy-preserving nature.

We also calculated the estimated disclosure protection score using SDMetrics^37^ summarized across biomedical datasets in Supplementary Material Table S3. This metric describes how difficult it is for an attacker to correctly guess sensitive information based on knowing certain attributes using an algorithm called Correct Attribution Probability (CAP) and compares it to a baseline of randomly guessing sensitive information. We used the cancer column as sensitive and the columns in configuration 4 in Fig. 7 as known. For cancer-specific datasets with binary cancer columns, we calculated the scores using a subset of individuals without diagnosis to balance the random baseline.

## Supplementary information

Supplementary Information

## Data Availability

The datasets used in this study are derived from sensitive biobank data containing potentially identifiable health information. Due to ethical, legal, and data protection regulations, these data are not publicly available. The data were accessed under specific institutional and regulatory approvals and cannot be shared or redistributed by the authors. Access may be granted to qualified researchers through the relevant data custodians, subject to appropriate ethical approvals and data use agreements.

## Code availability

The code supporting this study will be updated and made publicly available at: https://github.com/vuhoangminh/SecureSynth-Framework.
