# Supplementary material for: Anonymization and visualization of health data and biomarkers
Source: NPJ Digit Med. 2026 May 2;9:347. doi: 10.1038/s41746-026-02662-x (PMC13135499; doi:10.1038/s41746-026-02662-x)
Supplement: Supplementary file 1 — Supplementary Information [file 41746_2026_2662_MOESM1_ESM.pdf]

Supplemental Material for "Anonymization and Visualization of Health Data and Biomarkers"

Minh H. Vu[1*], Daniel Edler[2], Carl Wibom[1], Martin Rosvall[2], Beatrice Melin[1]

[1]Department of Diagnostics and Intervention, Umeå University, Umeå, Sweden.
[2]Department of Physics, Umeå University, Umeå, Sweden.
[*]Corresponding author: minh.vu@umu.se.

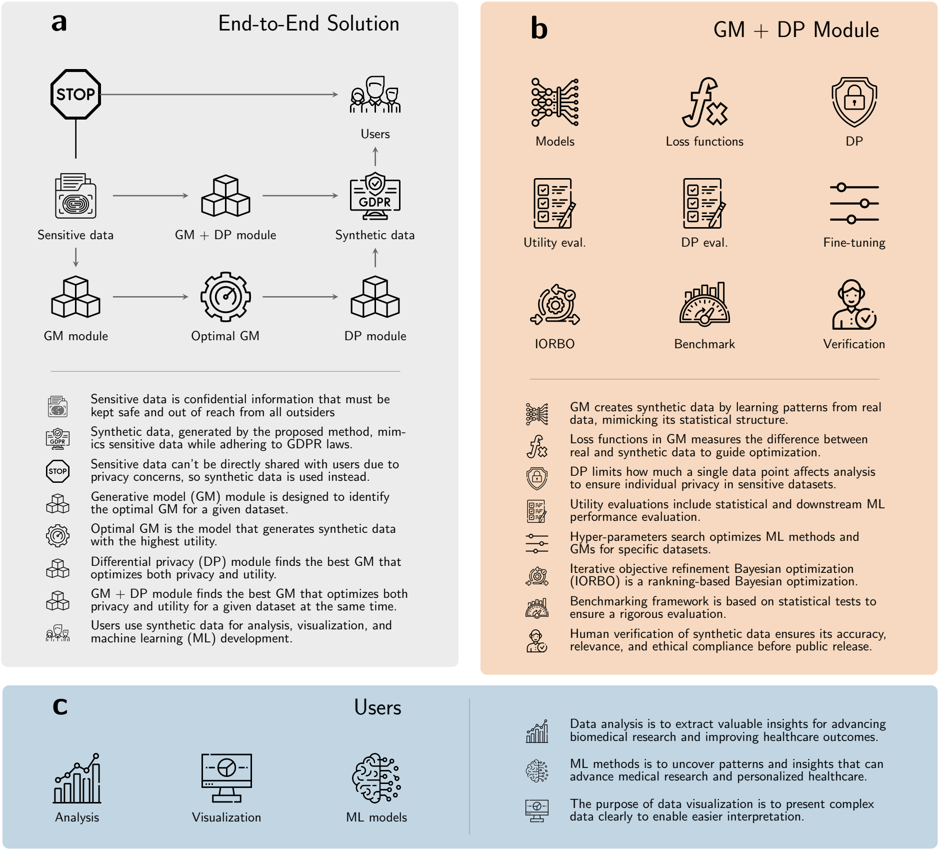

Fig. S1: **System architecture for end-to-end synthetic data generation, privacy enforcement, and downstream use**. **a,** End-to-End Solution: Sensitive data, protected by general data protection regulation (GDPR), cannot be shared directly with users. Instead, the deep generative model (DGM) and differential privacy (DP) modules generate synthetic data that mimics real data while ensuring privacy. The DGM module identifies the optimal DGM for a dataset, maximising utility, while the DP module selects a model that balances utility and privacy. The combined DGM + DP module finds the best model for a given dataset, enabling secure data sharing for analysis, visualization, and machine learning (ML). **b,** GM + DP Module: Generative models are configured with loss functions that measure differences between real and synthetic data. Privacy is enforced by assessing how individual data points affect synthetic datasets. Utility evaluations, including statistical and ML-based metrics, guide optimization using IORBO, a ranking-based Bayesian optimization method. Benchmarking ensures rigorous evaluation, while human verification ensures data accuracy, relevance, and compliance. **c,** Users: Synthetic data is used by researchers for biomedical analysis, ML development, and visualization, enabling extraction of valuable insights, advancement of personalized healthcare, and interpretable presentation of complex data.

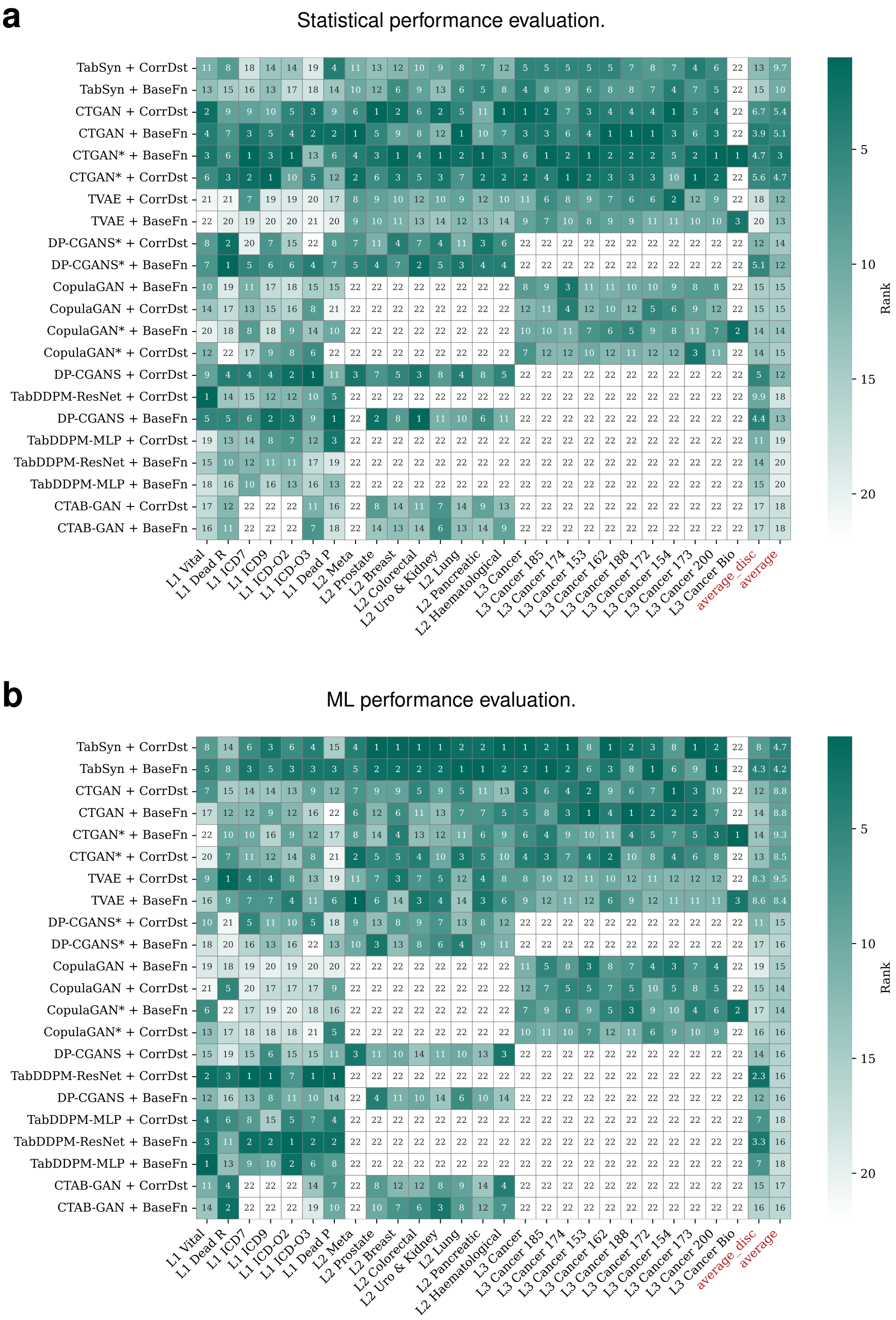

Fig. S2: **a,** Statistical and **b,** ML performance evaluation of DGMs across biomedical datasets.

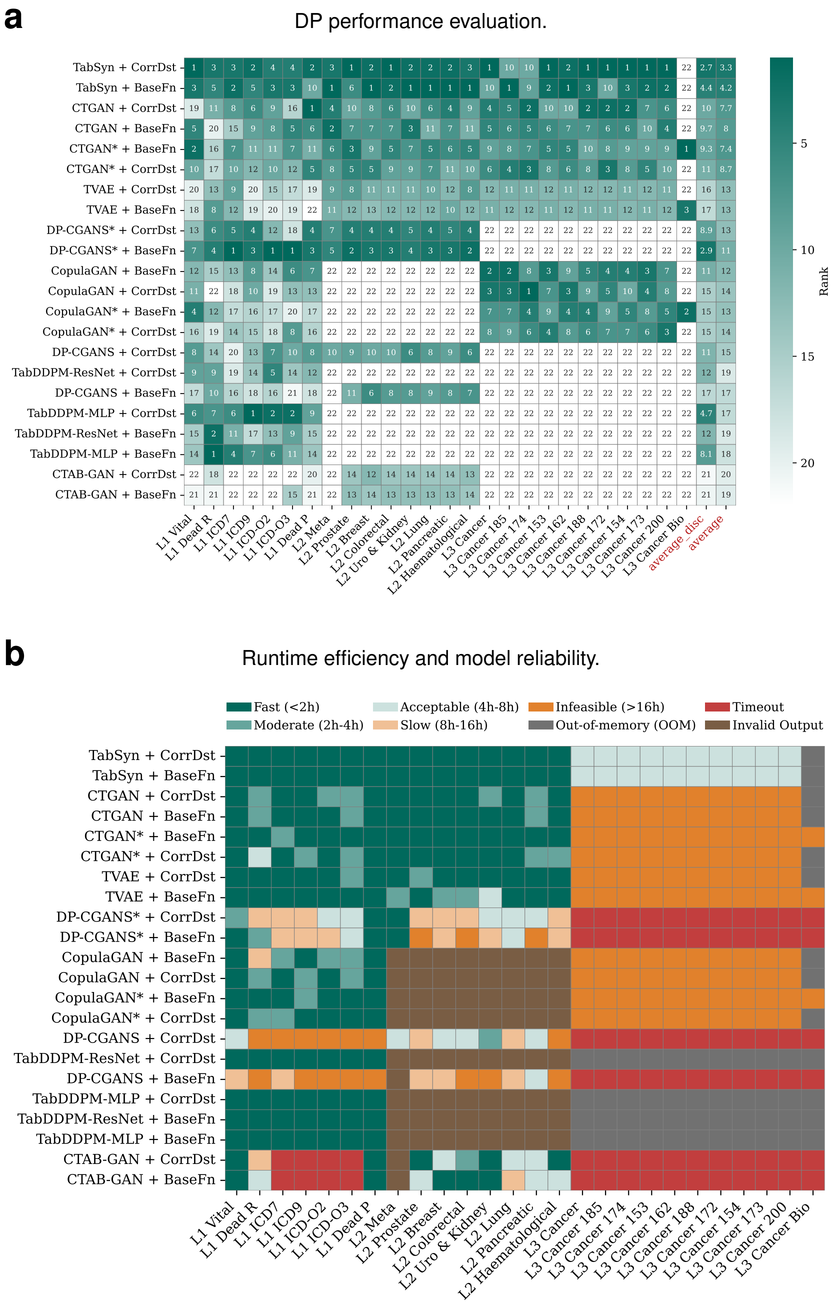

Fig. S3: **a,** DP performance evaluation and **b,** Runtime efficiency and model reliability across datasets. Heatmap in **b,** summarizes execution time categories (fast, moderate, acceptable, slow, infeasible) and failure modes (OOM, timeouts, invalid outputs) for evaluated generative models. `TabSyn`, `CTGAN`, and `TVAE` show consistent performance, while `DP-CGANS` and `CTAB-GAN` often fail on larger datasets.

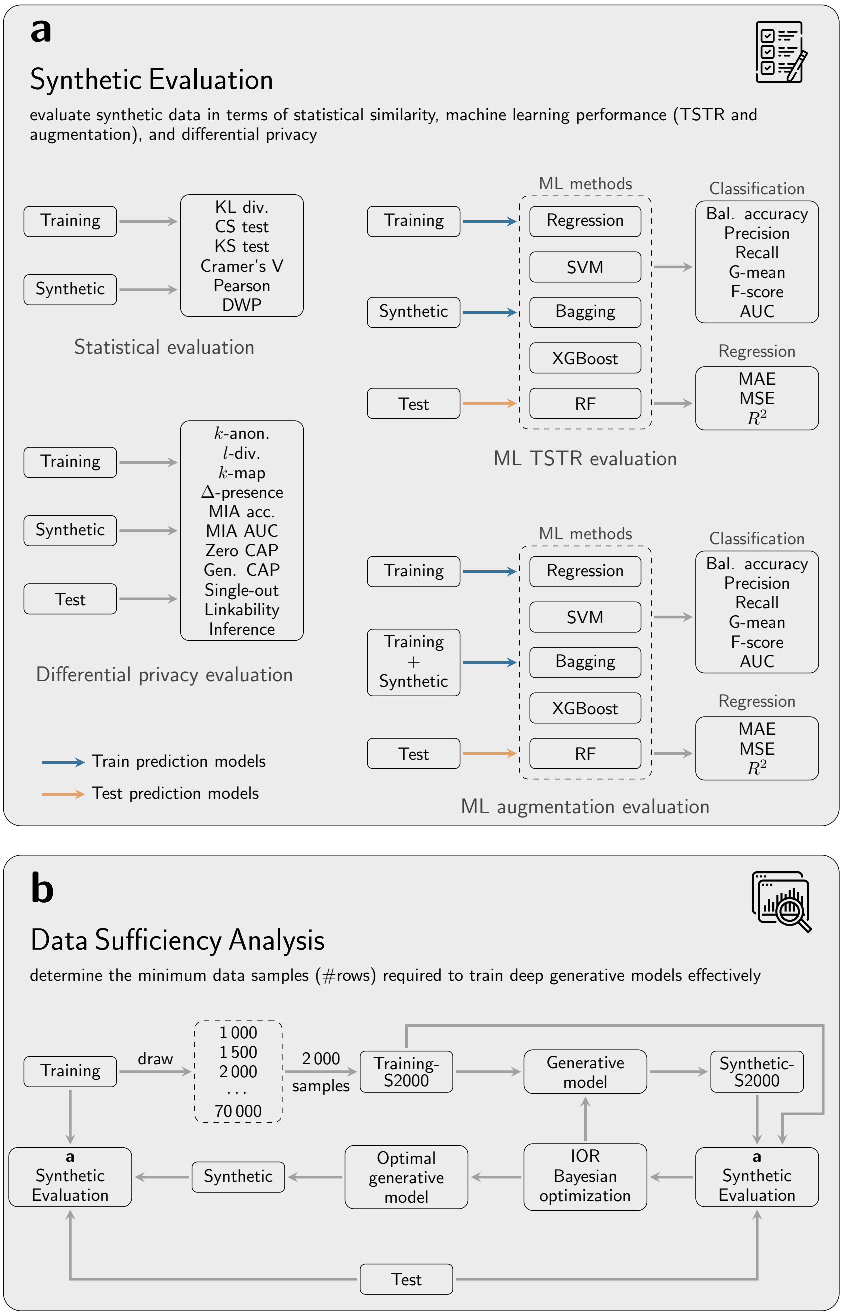

Fig. S4: **Overview of synthetic data evaluation and data sufficiency analysis for biomedical DGMs**. **a,** Synthetic Evaluation: Performance is assessed across statistical fidelity, ML-based TSTR, ML augmentation, and DP metrics. **b,** Data Sufficiency Analysis: Examination of the minimum sample sizes required to train DGMs that generate high-fidelity synthetic data in biomedical contexts.

Evaluation	Metric	TabSyn	CTGAN	TVAE	Direction
ML TSTR	regression_acc	0.253239	0.386514	0.148459	↓
ML TSTR	regression_precision	0.000369	0.000198	0.003644	↓
ML TSTR	regression_recall	0.000399	0.000024	0.152605	↓
ML TSTR	regression_f1	0.000269	0.000216	0.090305	↓
ML TSTR	regression_gmean	0.156597	0.546342	0.003713	↓
ML TSTR	regression_roc	0.042327	0.100868	0.006723	↓
ML TSTR	bagging_acc	0.257701	0.145459	0.163717	↓
ML TSTR	bagging_precision	0.000136	0.000332	0.003878	↓
ML TSTR	bagging_recall	0.000299	0.000223	0.152505	↓
ML TSTR	bagging_f1	0.000118	0.000332	0.090154	↓
ML TSTR	bagging_gmean	0.146309	0.386273	0.051327	↓
ML TSTR	bagging_roc	0.042325	0.101484	0.005416	↓
ML TSTR	xgboost_acc	0.259146	0.213628	0.626829	↓
ML TSTR	xgboost_precision	0.002759	0.003783	0.004815	↓
ML TSTR	xgboost_recall	0.000100	0.001022	0.155089	↓
ML TSTR	xgboost_f1	0.001543	0.002293	0.095617	↓
ML TSTR	xgboost_gmean	0.001659	0.569235	0.551078	↓
ML TSTR	xgboost_roc	0.043597	0.119722	0.040303	↓
ML TSTR	svm_acc	0.009140	0.000062	0.036161	↓
ML TSTR	svm_f1	0.001247	0.002159	0.856922	↓
ML augment	regression_acc	0.013212	0.206350	-0.114275	↑
ML augment	regression_precision	0.001131	0.000566	-0.002861	↑
ML augment	regression_recall	0.000200	0.000100	-0.000998	↑
ML augment	regression_f1	0.000672	0.000337	-0.001757	↑
ML augment	regression_gmean	-0.461712	-0.498711	-0.103423	↑
ML augment	regression_roc	0.006356	-0.016026	-0.005493	↑
ML augment	bagging_acc	-0.009019	0.172992	-0.183871	↑
ML augment	bagging_precision	0.000898	0.000898	-0.002728	↑
ML augment	bagging_recall	0.000100	0.000100	-0.000898	↑
ML augment	bagging_f1	0.000522	0.000522	-0.001645	↑
ML augment	bagging_gmean	-0.498708	-0.546342	-0.061214	↑
ML augment	bagging_roc	0.006579	-0.015554	-0.006277	↑
ML augment	xgboost_acc	-0.440868	-0.098804	-0.249808	↑
ML augment	xgboost_precision	-0.045715	-0.039335	-0.182884	↑
ML augment	xgboost_recall	-0.018766	-0.014674	-0.069974	↑
ML augment	xgboost_f1	-0.028848	-0.024386	-0.115225	↑
ML augment	xgboost_gmean	-0.236369	-0.174071	-0.169801	↑
ML augment	xgboost_roc	0.009122	-0.028245	-0.032802	↑
ML augment	svm_acc	0.002671	-0.013828	0.007178	↑
ML augment	svm_f1	-0.002529	-0.002207	0.000963	↑

Table S1: **Additional results on ML evaluations**. The ML TSTR metric reports the absolute difference between performance on synthetic and original data (↓: lower is better), while the ML augmentation metric evaluates improvements when augmenting real data with synthetic data (↑: higher is better). Results are shown for `TabSyn`, `CTGAN`, and `TVAE` on the `L3 Cancer` dataset.

Evaluation	Metric	TabSyn	CTGAN	TVAE	Direction
DP	k_anonymization_synthetic	391.000000	824.000000	999.000000	↑
DP	l_diversity_synthetic	391.000000	824.000000	999.000000	↑
DP	k_map	705.000000	199.000000	1.000000	↑
DP	delta_presence	1.380652	2.527638	1924.999981	↓
DP	re_identification_score	0.382740	0.035539	0.481719	↓
DP	domias_mia_accuracy	0.648924	0.648924	0.648924	↓
DP	domias_mia_auc	0.998315	0.999192	0.997629	↓
DP	categorical_zero_cap	1.000000	1.000000	1.000000	↑
DP	categorical_generalized_cap	1.000000	1.000000	1.000000	↑
DP	dcr_rf	6.438641	7.363734	6.043102	↑
DP	nndr_rf	0.884372	0.910310	0.896746	↑
DP	single_out_train_vs_control_4	0.003970	-0.000503	0.003458	↓
DP	single_out_train_vs_naive_4	-0.005954	-0.019848	-0.007939	↓
DP	single_out_risk_8	0.155187	0.046812	0.079965	↓
DP	single_out_train_vs_control_8	0.151275	0.045220	0.078131	↓
DP	single_out_train_vs_naive_8	0.154811	0.047634	0.073436	↓
DP	linkability_4	0.002199	0.000300	0.003300	↓
DP	linkability_5	0.002902	0.000699	0.004505	↓
DP	linkability_7	0.004016	0.001201	0.007030	↓
DP	linkability_8	0.004928	0.000601	0.008154	↓
DP	linkability_9	0.005344	0.002307	0.007672	↓
DP	linkability_10	0.006060	0.003314	0.009607	↓
DP	inference_high_bp	0.676147	0.614513	0.708645	↓
DP	inference_sm_status	0.313377	0.224041	0.363285	↓
DP	inference_sm_start	0.114979	0.069425	0.046131	↓
DP	inference_sn_status	0.583145	0.636919	0.552071	↓
DP	inference_sm_status_alt	0.328474	0.512275	0.102502	↓
DP	inference_pa_index	0.231311	0.215014	0.249656	↓
DP	inference_rand36_pf	0.290022	0.431513	0.256367	↓
DP	inference_rand36_rp	0.486442	0.329653	0.482053	↓
DP	inference_rand36_bp	0.124554	0.218584	0.197369	↓
DP	inference_rand36_sf	0.427925	0.333337	0.397133	↓
DP	inference_qol_d1	0.349315	0.368877	0.384407	↓
DP	inference_qol_d2	0.387783	0.386011	0.432189	↓
DP	inference_qol_d3	0.187719	0.219183	0.198614	↓
DP	inference_qol_d4	0.233134	0.194859	0.217282	↓
DP	inference_qol_d5	0.233093	0.222781	0.232702	↓
DP	inference_qol_d6	0.202458	0.206569	0.237835	↓
DP	inference_qol_d7	0.179557	0.199579	0.219684	↓
DP	inference_qol_d8	0.207000	0.216331	0.230492	↓
DP	inference_qol_d9	0.198129	0.217844	0.218520	↓
DP	inference_qol_d10	0.365163	0.388259	0.404496	↓
DP	inference_qol_d11	0.229959	0.229800	0.227626	↓

Table S2: **Additional results on data privacy (DP) metrics**. We report scores for synthetic datasets generated by `TabSyn`, `CTGAN`, and `TVAE` on the `L3 Cancer` dataset. Unless otherwise noted, all values follow the convention (↑: higher is better, ↓: lower is better).

Dataset	Quality Score	Column Shapes	Column Pair Trends	Diagnostic Score	Data Validity	Data Structure	Disclosure Protection Score
L3 Cancer	95	96	95	100	100	100	88
L3 Cancer Positive	92	95	90	99	99	100	88
L3 Cancer Binary	95	96	95	100	100	100	72
L3 Cancer Bio	68	77	59	99	99	100	97
L3 Cancer Bio Positive	68	77	58	97	94	100	97
L3 Cancer Bio Binary	68	78	59	99	99	100	100
L3 Cancer 185	95	96	94	99	99	100	77
L3 Cancer 174	95	96	95	99	99	100	86
L3 Cancer 153	95	96	94	99	99	100	92
L3 Cancer 162	95	96	94	99	99	100	93
L3 Cancer 172	95	96	94	99	99	100	100
L3 Cancer 185 Positive	91	94	88	99	99	100	-
L3 Cancer 174 Positive	89	92	86	98	97	100	-
L3 Cancer 153 Positive	89	93	84	99	98	100	-
L3 Cancer 162 Positive	86	90	82	99	99	100	-
L3 Cancer 172 Positive	88	93	83	99	99	100	-

Table S3: **Statistical quality and privacy evaluation using SDMetrics across biomedical datasets**. We include “Binary” versions replacing the multiclass cancer column with a binary, and “Positive” versions using the subset of individuals with a positive diagnosis. Scores formatted as percentages.
